# Bioactivity-Guided Synthesis: In Silico and In Vitro Studies of *β*-Glucosidase Inhibitors to Cope with Hepatic Cytotoxicity

**DOI:** 10.3390/molecules28186548

**Published:** 2023-09-09

**Authors:** Aneela Khushal, Umar Farooq, Sara Khan, Azhar Rasul, Tanveer A. Wani, Seema Zargar, Sohail Anjum Shahzad, Syed Majid Bukhari, Nazeer Ahmad Khan

**Affiliations:** 1 Department of Chemistry, COMSATS University Islamabad, Abbottabad Campus, Abbottabad 22060, Pakistan; aneelakhan460@gmail.com (A.K.); sashahzad@cuiatd.edu.pk (S.A.S.); majidbukhari@cuiatd.edu.pk (S.M.B.); maliknazeerdik@gmail.com (N.A.K.); 2 Beijing National Laboratory for Molecular Sciences, State Key Laboratory of Molecular Reaction Dynamics, Institute of Chemistry, Chinese Academy of Sciences, Beijing 100190, China; 3 Department of Zoology, GC University Faisalabad, Faisalabad 38000, Pakistan; drazharrasul@gmail.com; 4 Department of Pharmaceutical Chemistry, College of Pharmacy, King Saud University, P.O. Box 2457, Riyadh 11451, Saudi Arabia; twani@ksu.edu.sa; 5 Department of Biochemistry, College of Science, King Saud University, P.O. Box 22452, Riyadh 11451, Saudi Arabia; szargar@ksu.edu.sa

**Keywords:** β-glucosidase, phthalimide, phthalamic acid, HepG2 cell line, hepatic cytotoxicity, molecular docking, MD simulations

## Abstract

The major cause of hyperglycemia can generally be attributed to β-glucosidase as per its involvement in non-alcoholic fatty liver disease. This clinical condition leads to liver carcinoma (HepG2 cancer). The phthalimides and phthalamic acid classes possess inhibitory potential against glucosidase, forming the basis for designing new phthalimide and phthalamic acid analogs to test their ability as potent inhibitors of β-glucosidase. The study also covers in silico (molecular docking and MD simulations) and in vitro (β-glucosidase and HepG2 cancer cell line assays) analyses. The phthalimide and phthalamic acid derivatives were synthesized, followed by spectroscopic characterization. The mechanistic complexities associated with β-glucosidase inhibition were identified via the docking of the synthesized compounds inside the active site of the protein, and the results were analyzed in terms of the best binding energy and appropriate docking pose. The top-ranked compounds were subjected to extensive MD simulation studies to understand the mode of interaction of the synthesized compounds and binding energies, as well as the contribution of individual residues towards binding affinities. Lower RMSD/RMSF values were observed for **2c** and **3c**, respectively, in the active site, confirming more stabilized, ligand-bound complexes when compared to the free state. An anisotropic network model was used to unravel the role of loop fluctuation in the context of ligand binding and the dynamics that are distinct to the bound and free states, supported by a 3D surface plot. An in vitro study revealed that **1c** (IC_50_ = 1.26 µM) is far better than standard acarbose (2.15 µM), confirming the potential of this compound against the target protein. Given the appreciable potential of the candidate compounds against β-glucosidase, the synthesized compounds were further tested for their cytotoxic activity against hepatic carcinoma on HepG2 cancer cell lines. The cytotoxicity profile of the synthesized compounds was performed against HepG2 cancer cell lines. The resultant IC_50_ value (0.048 µM) for **3c** is better than the standard (thalidomide: IC_50_ 0.053 µM). The results promise the hypothesis that the synthesized compounds might become potential drug candidates, given the fact that the β-glucosidase inhibition of **1c** is 40% better than the standard, whereas compound **3c** holds more anti-tumor activity (greater than 9%) against the HepG2 cell line than the known drug.

## 1. Introduction

The β-glucosidase is known to get involved in the regulation of several biological processes, including, but not limited to, the immune system, biological transformations, and the catabolism of glucosidase in the human tissues. This enzyme has the capacity to convert various saccharides to glucose units [1,2] and, as a result, can lead to non-alcoholic fatty liver disease (NAFLD). This disease is present across the globe and has affected 20 to 30% of the adult population [3]. NAFLD is sometimes misdiagnosed as simple steatosis (fat accumulation in the liver; benign with decent prognosis). Non-alcoholic steatohepatitis (NASH) is a phase of NAFLD steatosis, which is associated with hepatocellular disintegration, inflammation, and fibrosis [4]. In almost 10% of NASH patients, fibrosis deteriorates and progresses to cirrhosis, a situation characterized by irretrievable scarring and hardness of the hepatic tissue, thereby preventing it from its normal functioning [5]. Although the exact reason for the change from basic steatosis to NASH is unknown, the in vitro and in vivo studies project that chronic hyperglycemia-induced hepatocyte apoptosis is highly significant throughout this transition. Moreover, epidemiological research suggests that a decrease in glucose tolerance in patients with NAFLD is a predictor of NASH [6,7]. As a result, it is critical to comprehend the role and mechanism of hyperglycemia in the induction of hepatocellular degeneration. Hepatocyte apoptosis is thought to be caused by hyperglycemia-induced mitochondrial malfunction, which includes abnormalities of the mitochondrial electron (e^−^) transport chain, the excessive generation of reactive oxygen species (ROS), and the impairment of membrane potential [8]. Mitochondrial failure can cause the leakage of cytochrome c into the cytosol, where it forms a complex with Apaf-1, recruiting and activating procaspase-9 into active caspase-9 [9,10,11]. NAFLD, in certain serious circumstances, has the tendency to translate itself into lethal liver carcinoma, which is one of the most prevalent malignant tumors in the world [12]. This results in liver malfunction, and the patients often have poor tolerance to chemotherapy treatments [13]. Therefore, highly effective medications for the eradication of hepatoma cells are urgently needed [14]. Various heterocyclic compounds possessing cytotoxic potential have been reported in this regard.

Among all N-substituted imines, the phthalimide and phthalamic acid derivatives are recognized as one of the most efficient skeletons. Due to being neutral and hydrophobic, these can easily pass through living membranes. Based on their compatibility with living cells, N-substituted phthalimides have a wide range of in vivo biological applications [15]. Phthalimide and phthalamic acid derivatives are used as hypolipidemic agents as these can decrease plasma cholesterol as well as triglyceride levels [16]. Isoindoline-1,3-dione-based drugs are particularly used as anti-cancer agents [17], anti-inflammatory compounds [18], ligands for D2 receptors [19], medicines to treat analgesic infections [20], neuroprotective drugs [21], fungicides [22], and anti-tuberculosis drugs [23]. They are also used as a catalyst [24] to synthesize various herbicides^25^. Therefore, a new series of phthalimide and phthalamic acid analogs have been designed, synthesized, and biologically evaluated by in vitro and in silico studies to reveal their β-glucosidase inhibition potential as well as their cytotoxic capacity.

## 2. Synthesis and Characterization

A series of compounds (**1c**–**12c**) were synthesized by using the reaction of substituted amines with phthalic anhydride in the presence of acetic acid. The structural elucidation of the synthesized compounds (**1c**–**12c**) was carried out by using ^1^H-NMR and ^13^C-NMR spectroscopic techniques. In the ^1^H-NMR analyses of the compound (**1c**–**4c**) under discussion, there is the band of two –OH protons at positions 9 and 19, which give singlet signals at δ 12.90 ppm, and an NH group at position 20 also gives a singlet at δ 10.65. Eight aromatic protons appear at δ 7.5–7.91. In ^13^C-NMR analysis, there appear two characteristic signals of carbonyl groups belonging to two carboxylic acids and one amide at δ 167.38, 167.96, and 167.07, respectively. The rest of the carbon atoms appear in the range of δ 118.81–143.65 ppm. In the ^1^H-NMR of compounds (**5c**–**12c**), aliphatic protons appear in the range of 2.34–3.70 ppm, and aromatic protons appear in the range of 7.26–7.90 ppm. The ^13^C-NMR analysis shows that the carbonyl carbons belonging to carboxylic acid and the amide group appear at δ 168.6, 167.9, and 166.9, respectively. All aromatic carbons appear in the range of δ 123.1–138.4, and all aliphatic carbon atoms appear in the range of 34.9–55.1 ppm.

### Structure Optimization of Synthesized Compounds

**Figure 1:**
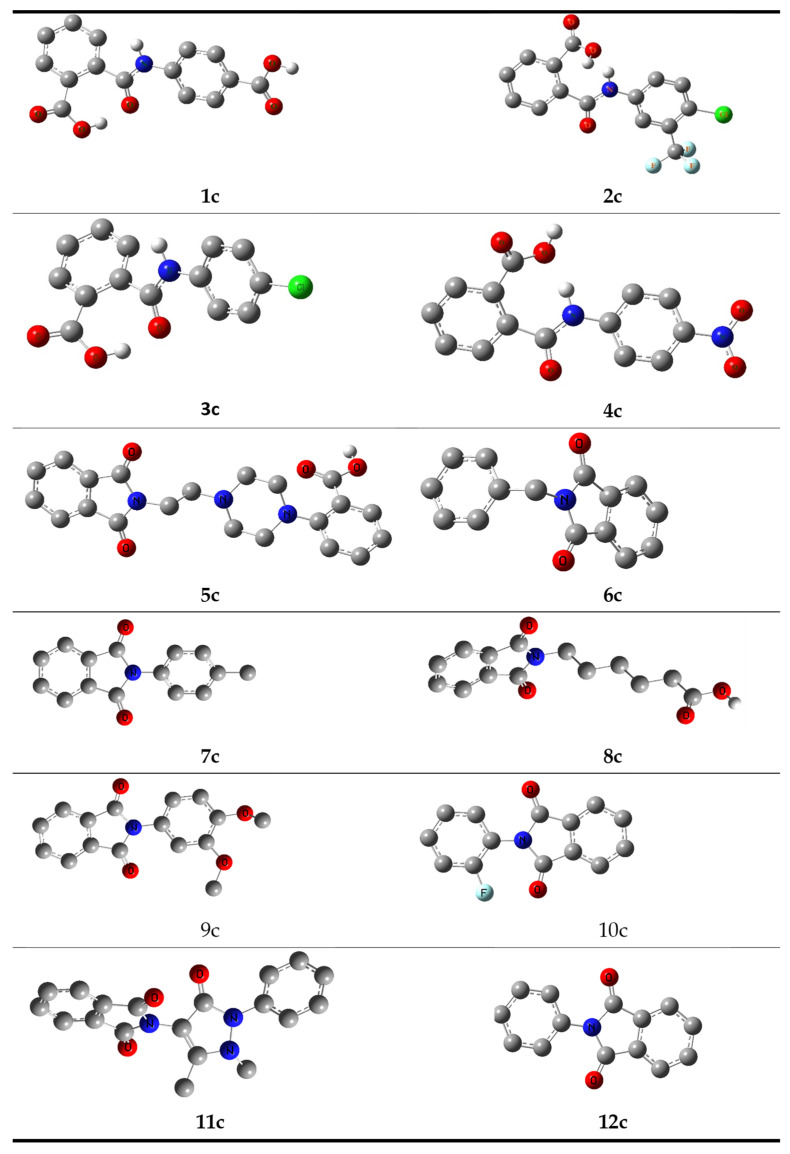
Optimized structures of compounds **1c**–**12c** using Gaussian 09. Ball and stick model of the compounds: white represents hydrogen, grey represents carbon, red represents oxygen, blue represents nitrogen, and cyan represents fluorine.

DFT study was used to optimize the structure of synthesized compounds using Gaussian 09 by employing the B3LYP method with a 6–31G basis set (Figure 1).

## 3. Molecular Docking Study

A molecular docking study was performed on the optimized structures of compounds **1c**–**12c** to unravel the potentiality of these synthesized compounds to occupy the active pocket in a way that would disrupt the hydrolysis of the glycosidic bond, leading to the inhibition of β–glucosidase activity. The docking of compounds **1c**–**12c** was carried out using MoE, and the docked complexes were analyzed in terms of their binding interactions inside the active pocket. It was observed that the existence of compounds **1c**–**12c** inside the pocket is governed by both electrostatic interactions and Lenard Johns van der Waal’s potential energy, with several target residues comprising the active pocket of the receptor. The docking of compounds within the active pocket resulted in the generation of multiple poses for each compound. The binding energy coupled with the most acceptable RMSD results (closeness to the native ligand position within the active pocket) resulted in the exclusion of rest from the list, whereas the complexes of compounds **1c**–**12c** were subjected to extensive MD simulation study.

### 3.1. ADME Analysis

The in silico ADME characteristics of the compounds were determined by using proTox II, a free website (https://tox-new.charite.de/ 2022). The bioavailability radar’s colorful zone reflects the best optimal region for oral bioavailability. The chemicals’ bioavailability radar pictures indicate that they are suitable for bioavailability. The substances have a high GI absorption, according to their calculated ADME characteristics (Table S2).

### 3.2. MD Simulations Study—Global Stability Indices

**Figure 2:**
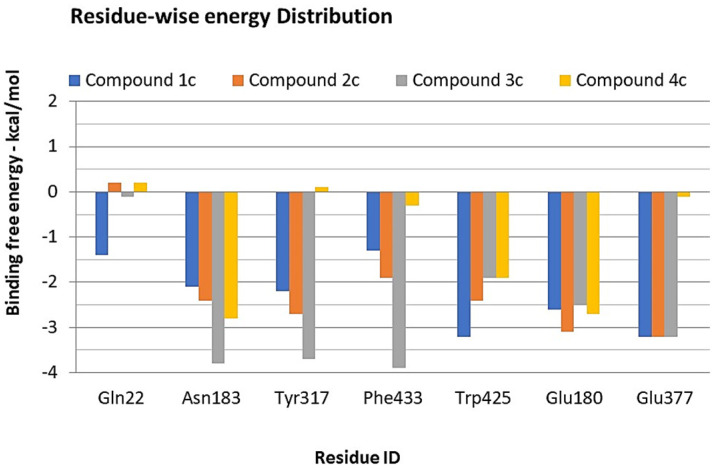
Residue-wise distribution of energy; percentage retention of inhibitors inside the active pocket.

**Figure 3:**
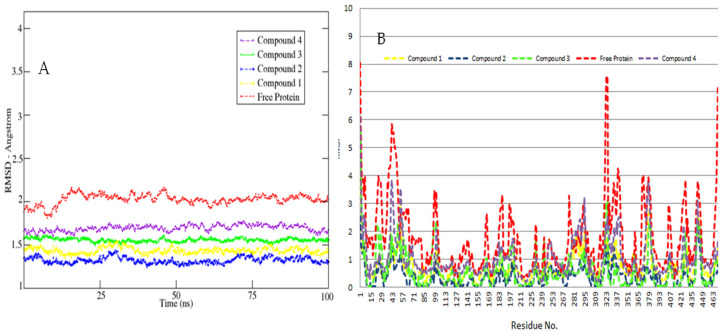
(**A**) Global stability analysis—RMSD of free protein and protein-ligand complexes for a time period of 100 ns each. (**B**) RMSF graph of ligand-protein complexes vs. free protein: the complexes are color-coded as **1c**: yellow, **2c**: blue, **3c**: green, and **4c**: purple. RMSD/RMSF for free protein is color-coded red.

The global stability indices (RMSD and RMSF) provide a better picture of the mechanical complexities associated with receptor-ligand binding in the given time scale (Figure 2 and Figure 3). The root mean square deviation (RMSD) of the protein Cα atoms as a function of time with respect to the initial position was used to monitor the simulations. Protein RMSD in the ligand-bound system grew to about 1.4–1.7 Å and stabilized during the initial 5–10 ns of unrestrained simulations, whereas for the free protein system, it resonated near 2.2 Å. Lower RMSD values for compounds in their respective protein-ligand complexes would imply sufficient ligand retention within the active pocket during the explored timescale. For each ligand-bound protein, the root mean square fluctuation per residue with respect to the free state was calculated in order to acquire a deeper understanding of the stability of the complex binding site. Information on the role of specific protein residues in the structural fluctuations of the ligand/protein complex is provided by this flexibility validation criterion.

In comparison to the initially optimized structure, the RMSF determines the average deviation for each residue from its reference point over time. An RMSF cut-off value of 0.25 Å was assigned to determine whether the structural movements had significantly changed, and the residues with values higher than 0.25 Å were more flexible. The RMSF data from the ligand-free protein revealed high RMSF values for the terminal residues, as well as the loop regions, when compared to the bound state. A different fluctuation pattern was observed in the ligand-bound complexes. Lower RMSF values were exhibited by **2c**, **3c**, and **5c**, which are vicinal residues and C-terminal-free residues, respectively. The proximity of the receptor pocket residues to the protein terminal side results in more stable ligand-bound complexes than in the free state, according to these studies. Lower RMSF values were assigned to **3c** at the N-terminus when compared to **1c** and **2c**, which suggests that N-terminal-free residues and vicinal residues may affect 3c protein binding through MD simulation. These subsequent residues may be strongly associated with the stability of the compounds, following the substantial conformational/orientational shift beyond 20 ns and up to 70 ns in the MD simulation run since they are located far from the reference binding site. The literature reveals that Glu180, Glu377, Trp245, and Tyr433 are the active site residues where the substrate binds and promotes the activity of β-glucosidase; therefore, the lower amplitude fluctuation on and around these regions confirms the accommodation of ligands inside the active pocket [25].

### 3.3. Hot Spot Regions in Binding

**Figure 4:**
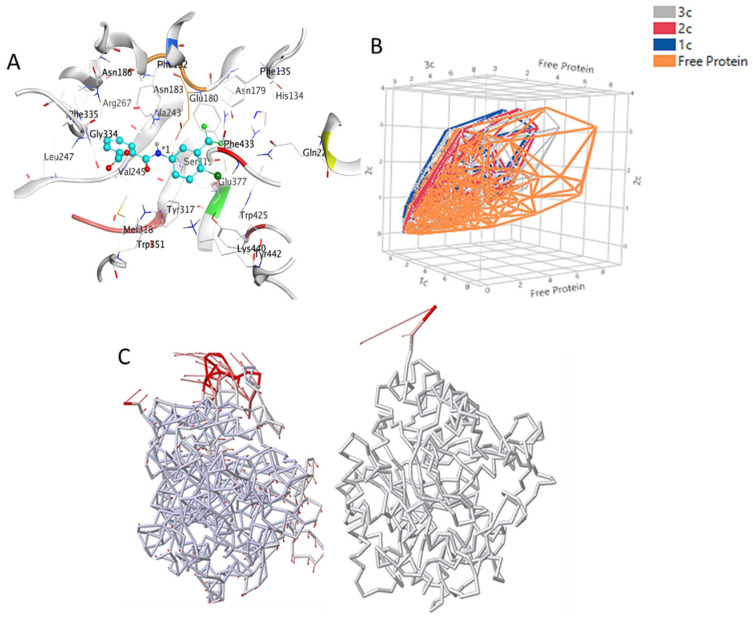
(**A**) Close-up image of the simulated binding pocket of β-glucosidase complexed with **3c**. The compound is shown as blue in the ball and stick representation. The different fragments are shown as grey, whereas the critical residues are color-coded differently. Loop L1 is shown in orange, L2 in red, Glu377 in green, Gln22 in yellow, and Phe433 in blue. (**B**) The surface plot of the dynamics of the bound protein when compared to the free protein. Color code: blue for **1c**, red for **2c**, grey for **3c**, and orange for free protein. (**C**) The 3D dynamics of the ligand-bound vs. free protein simulations of the bound receptor in comparison to free β-glucosidase provide a detailed atomistic picture of inhibitor binding in a near-native environment. The simulation gives information on inhibitor binding dynamics and allows us to characterize the binding pocket in greater depth.

The crystal structure of β-glucosidase reveals a number of key residues that encapsulate the binding pocket and are likely to contribute towards competitive inhibitor binding [26]. These include the residues in active loop L1 (Glu180, Asn183—color-coded brown), Phe192 (helix, color-coded blue), active loop L2 (Tyr317, Met318, color-coded orange), and Glu377 (green) in the sheet of the target protein, and two residues (Trp425 and Phe433) in the loop 1 and 2 linker region (see Figure 4 for the locations of these residues). We executed 100 ns of MD simulations for the ligand-free and bound complexes.

An anisotropic network model (ANM) was used to investigate the collective motions of the ensemble of the structures obtained because of the MD simulations for β-glucosidase with its bound and free state. In particular, a large fluctuation of the loop domain and its allosteric role is discussed in the context of protein function. The dynamics observed as a result of the anisotropic network model suggest that lid is the only region of the protein that shows distinct functional mobility, suggesting a more rigid framework in the bound state when compared to the free state (Figure 4).

The β-glucosidase-binding pocket is wide and flexible, located in the extracellular collar of the receptor, which is a critical area that undergoes major structural changes during binding following loop closure. The residues facilitating the binding of compounds **1c**–**3c** within the active site are given in Table S2. The data indicate that a number of amino acids consistently aid in the binding of compounds **1c**–**3c** and **5c** within the active pocket, albeit the contributions made by each of these residues vary depending on how the substance is oriented within the active pocket. In essentially all of the binding poses of all three inhibitors, residues Glu180, Glu377, Tyr317, Asn183, Phe433, Val245, and Gln22 create strong interactions with the compounds, with appreciable binding energy computed via residue-wise distribution of energy (Figure 4).

### 3.4. Binding Free Energy Calculations—MMPBSA Approach

The molecular docking study revealed the binding of all four compounds in the active pocket with varying binding energies and poses, facilitated by the varying interactions experienced by each compound inside the active pocket. Alternatively, the binding energies of the compounds **1c**–**3c** and **5c** were computed using the MMPBSA approach (Table 1). Compound **5c** showed a binding energy of −14.34 kcal/mol, which was supported by the residues encapsulating the active pocket. The key distributions for the binding were made by Glu180, Trp425, and Asn183 via hydrogen bonds and hydrophobic interactions. All the analyses of the MMPBSA binding energy calculations and simulated poses were performed on the last 85 ns of a stable portion of the trajectories. Those residues that remained within 4 Å of a ligand for over 60% of the analysis period were considered to be in contact with the ligand in its simulated pose. Unsurprisingly, the docking of compounds in the active pocket resulted in a lower number of alternative binding modes when compared to MD simulations. The docking scores of the receptor compound complexes **4c** and **6c**–**12c** are lower than those of **1c**–**3c** and **5c**. The compounds (**1c**–**3c**, **5c**) docked inside the active pocket remained stable during the entire MD simulation trajectory period, inferring that the binding of the compounds inside the active pocket resulted in the stabilization of the binding pocket.

### 3.5. Interaction Pattern of Compounds ***1c***–***3c*** and ***5c*** within the Binding Pocket

Compounds **1c**–**3c** and **5c** formed multiple non-electrostatic contacts and a few hydrogen bonds with the active site residues of the receptor. The binding score of compound **1c** with β-glucosidase turned out to be −18.23 kcal/mol. The NH-group in the five-membered ring of Trp425 binds with a hydrogen bond acceptor by means of the -OH group of carboxylic acid, which is present at the para position of compound **1c**, with a bond distance of 2.37 Å. Gln22 is a highly conserved amino acid in all glucosidases and is involved in hydrogen bond formation with glucose at subsite 1, making it an important catalytic residue. The NH group of the amino acid residue Gln22 interacts as a hydrogen bond acceptor with the oxygen atom of the benzoyl moiety, with an average distance of 2.25 Å. Glu180 and Glu377 are the critical active site residues facilitating the cleavage of the glycosidic bond, with the former being a proton donor and the latter acting as a nucleophile. The oxygen atom of Glu180 shows a hydrogen bond (2.59 Å) with the NH group of phthalimide. The Glu377 interacts with the π-bond of the ring B residues, presenting a bond distance of 4.89 Å either through π –ion interaction or electrostatic interactions. The phe433 and try317 show a π–π stacked interaction with the benzoyl group of the ring B structure, with bond distances of 4.77 and 3.92 Å, respectively. On the other hand, the π-bonds of the ring A residues interact with the alkyl groups of Ala243 (sheet) and Val245 at bond distances of 4.84 and 3.72 Å, respectively (Figure 2).

The binding score of compound **2c** with β-glucosidase is −17.56 kcal/mol. The Asn183 of L2 is another conserved residue across the glucosidase family. The NH-group of Asn183 interacts with the carbonyl oxygen in phthalamic acid as a hydrogen bond donor, with a bond distance of 1.98 Å. The π-bonds of the amino acids Try317 and Phe433 of the L1 and L2 linker region interact with the nitrogen atom of NO_2_ as π-cation forces, with a bond distance of 4.24 and 4.46 Å, respectively. The attractive charges, i.e., electrostatic interactions, are shown by the oxygen atom of Glu377 (catalytic residue), Glu180 (catalytic residue—L1), and SO_2_ (cofactor), with the nitrogen atom of NO_2_ presenting bond distances of 5.15, 4.61, and 5.25 Å, respectively. The π bond of Try317 (loop) shows π–anion interaction with the oxygen atom of NO_2_, with a bond distance of 4.24 Å. Phe192 interacts through π–π stacked interactions with the benzoyl moiety of the ring B residues and shows a bond distance of 5.69 Å. With bond distances of 4.24 and 5.29 Å, respectively, the π-bonds of ring residue A interact with the alkyl groups of Val245 and Met318 (L2), respectively.

**Figure 5:**
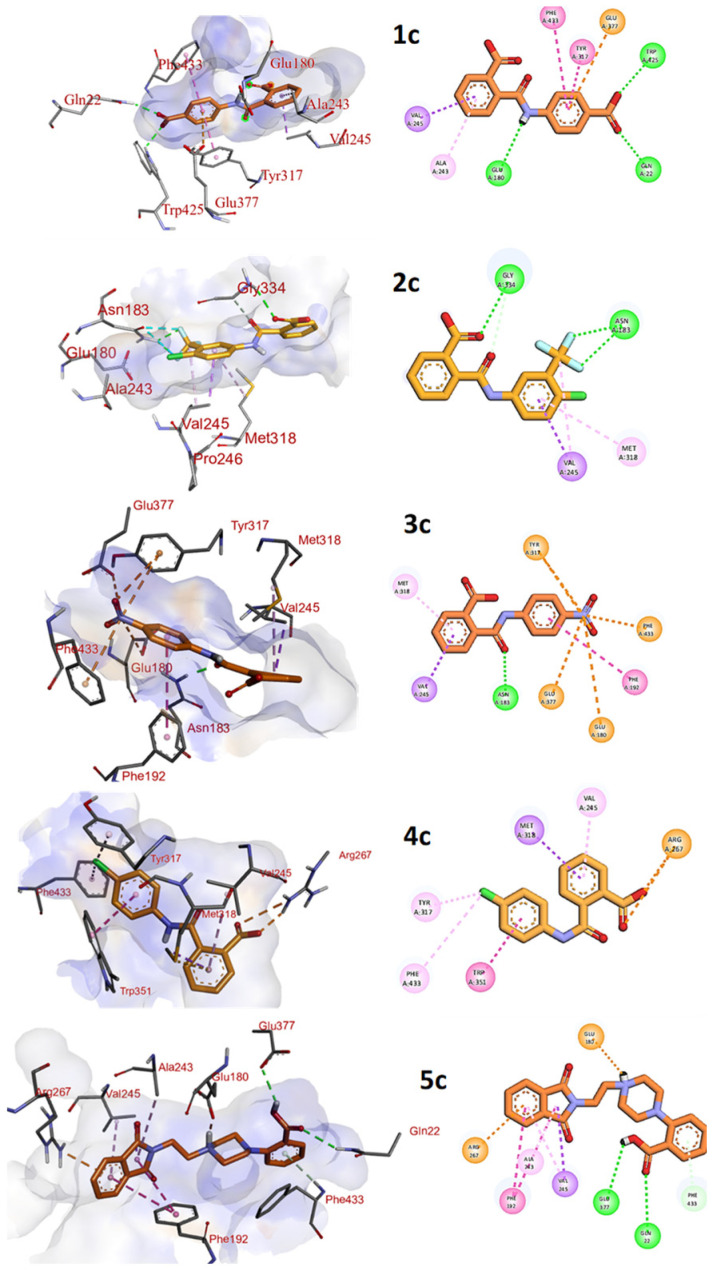
A stable receptor ligand poses with the lowest binding energy, presenting 2D and 3D binding interactions of the synthesized compounds with open and closed chains with β-glucosidase. All residues interacting within 4 Å of the ligands in the simulated structure are shown.

The binding score of compound **3c** with β-glucosidase is −20.07 kcal/mol. Strong halogen-oxygen (C-X–Y) interactions were observed among catalytic Glu180 and Glu377 with the fluorine of **3c**. The oxygen atom of catalytic Glu180 interacts with two fluorine atoms attached on **3c**, with bond distances of 3.05 Å and 3.35 Å, whereas Glu377 also shows strong O-H interactions with the fluorine atom on CF_3_, with a bond distance of 3.02 Å. It has been reported previously that the presence of the C-X–Y bond improves the total binding affinity of the drug. Additionally, to induce the therapeutic effect, the first-hand interaction of the drug takes place with biopolymers that reside in an aquatic environment. Halogen bonds would have considerably more intricate physiological functions than is now understood [27]. The amino acid residues Trp245, Tyr317, and Phe135 show π–π interactions with the compound **3c**, with bond distances 5.32, 5.43, and 4.97 Å. π–alkyl interactions were observed between Val245, Phe433, Phe192, and compound **3c**, with bond distances of 3.90, 5.14, and 5.76 Å, respectively. The hydrogen atom of Gly334 interacts with the oxygen atom of the carboxylic group, with a bond distance of 2.51 Å. When taken together, all these interactions in different proportions facilitate the retention of ligands inside the active pocket of the receptor atom (Figure 5).

### 3.6. In Vitro Studies

#### 3.6.1. β-Glucosidase Inhibition

**Figure 6:**
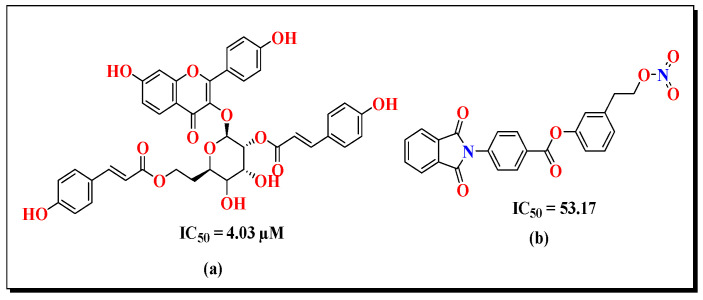
Structures and IC_50_ values of the reported compounds against (**a**) β-glucosidase and (**b**) HepG2 cell lines.

**Figure 7:**
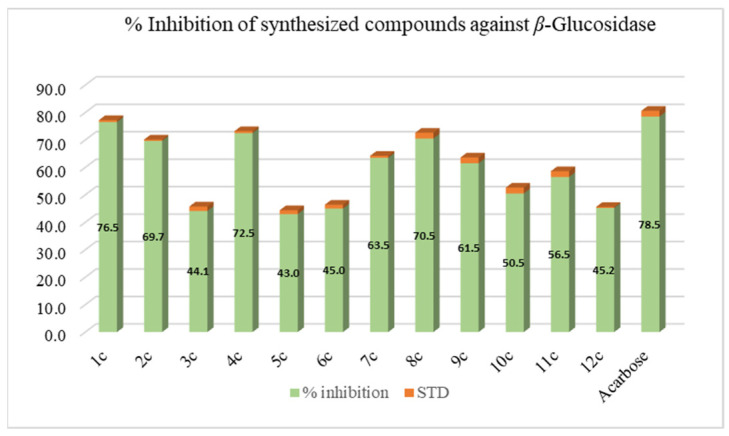
Percentage inhibition and standard deviation of synthesized compounds against β-glucosidase.

β-glucosidase inhibition activity of the synthesized compounds, with an enzyme concentration of 1.5 mg/mL were used for the inhibition assay. The IC_50_ values were determined at different concentrations using Graph Pad Prism v5.0. Notably, three compounds exhibited significant β-glucosidase inhibition, with IC_50_ values of 1.26 ± 0.23 µM, 1.26 ± 0.11 µM, 2.17 ± 0.11 µM, and 3.00 ± 0.17 µM for compounds **1c**, **5c**, **2c**, **3c**, **7c**, and **9c**, respectively (see Figure S6). Importantly, compounds **1c** and **5c** demonstrated superior inhibition compared to the standard compound (Acarbose), with an IC_50_ value of 2.15 ± 0.16 µM. In fact, compounds **1c** and **5c** exhibited nearly twice the activity against the enzyme when compared to the standard. Compounds **2c** and **3c** also displayed an inhibition potential similar to that of the reference compound, as depicted in Figure 6a [28]. The %inhibition and %viability against the HEPG2 cancer cell lines for all the compounds are given in Figure 7.

#### 3.6.2. %Viability of the Synthesized Compounds against Human HepG2 Hepatic Cancer Cells

**Figure 8:**
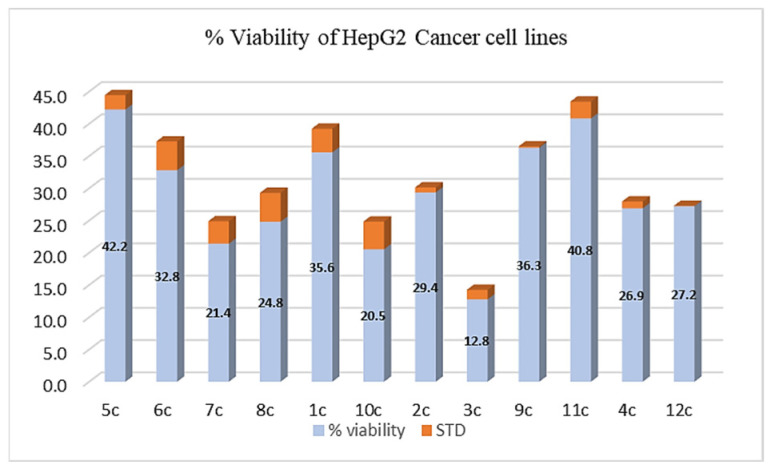
Percentage viability and standard deviation of the synthesized compounds against HepG2 cancer.

The viability of human HepG2 hepatic cancer cells, when exposed to the synthesized compounds, is summarized in Figure 8. Notably, compound **3c** emerges as being exceptionally potent against human HepG2 liver cancer cells. The other compounds, namely **3c**, **7c**, and **10c**, also exhibit strong responses, with %viabilities of 12.77%, 21.45%, and 25.55%, respectively, at a concentration of 200 µg. These compounds effectively target a specific site and effectively hinder the growth of cancer cells. In particular, compound **3c** stands out by having the most significant response against human HepG2 liver cancer cell lines, displaying a %viability of 12.77% ± 1.47% and an IC50 of 4.8 µM ± 0.16 µM (calculated using Graph Pad Prism software), as illustrated in Figure S7. In light of these findings, it can be concluded that the newly synthesized compounds are highly potent molecules in comparison to those reported previously. Moreover, these compounds surpass the standard drug thalidomide in inhibiting the HepG2 cell line. In fact, compound **3c** is considered even more potent than the reported analog of phthalamic acid, as demonstrated in Figure 6b [29].

### 3.7. Structure Activity Relationship (SAR)

**Figure 9:**
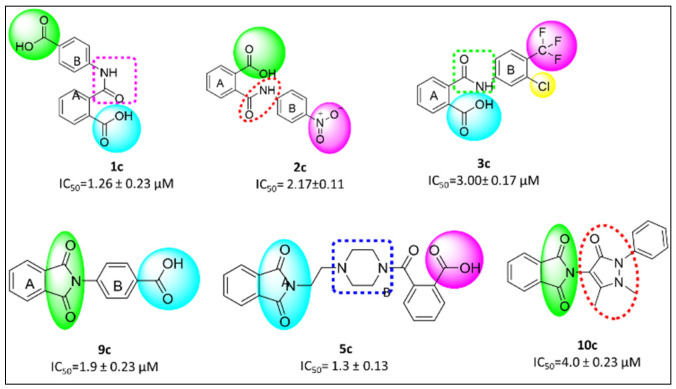
Structures and IC_50_ values for synthesized compounds against β-glucosidase.

SAR refers to the relationship between the chemical structure and the biological activity of a compound. In this study, β-glucosidase inhibition against the cancer (HEPG2) cell line depends on both the nature of the compound and its functional groups/substituent attached by a phenyl ring. For one series of phthalimide compounds comprised of an amide (NH–C=O) with an open chain and having a carboxylic acid group as the major common group, the substituted phenyl ring varies, affecting the biological activity of the compounds. The most potent compounds, in which the acidic groups (–COOH) are at an ortho-position in ring A (similarly, the same group at the para position of ring B), are responsible for the good inhibition of compound **1c**, and amide linkage is also responsible for the activity of compound (**1c**) (Figure 9). Compound **2c**, which has an IC_50_ = 2.16, is less potent than **1c** because the carboxylic acid group on the substituent is replaced with the nitro(–NO_2_), and when the carboxylic acid groups were replaced by halogen atoms, its activity decreased, i.e., when the para-substituted COOH replaced by the CF_3_-group at meta position Cl-atom was added, its activity decreased; furthermore, when the CF_3_-group was replaced by an H atom, this compound showed less potency to inhibit β-glucosidase. 

Another series of compounds has been synthesized, which have closed-chain phthalimides (**5c**–**12c**) as a major group and have various substituents at their N-position; this substituent varies their potency to inhibit β-glucosidase. Some of them are aliphatic or cyclic and some of them are aromatic when used with various substituents. Among the second series of compounds, compound **5c** exhibited excellent inhibition against β-glucosidase due the presence of the carboxylic acid (–COOH) group at the ortho position of ring B and the two carbonyls (amide-carbonyl color-coded blue) at ring A; the two nitrogen atoms of the six-membered ring of the piperazine moiety also enhance the potency of the compound **5c**, as show in figure. The aliphatic chain (either a long chain with a carboxylic acid moiety or a phenyl ring) exhibited low potency against β-glucosidase. This series of compounds with a phenyl ring that has various substituents exhibit more potency than others. A substituent on the ring effects the potency of the compounds, and the most active substituents have a carboxylic group as their major substituent. Although compound **9c** and **1c** have similar structures, **1c** has two carboxylic acid groups, and **9c** has one carboxylic acid group and two amide linkages, and this is why its activity decreases against a selected target. When compound **10c** has three carbonyl groups in its structure, its activity increases for the rest of the compound, but this is still less potent than the carboxylic acid groups.

**Figure 10:**
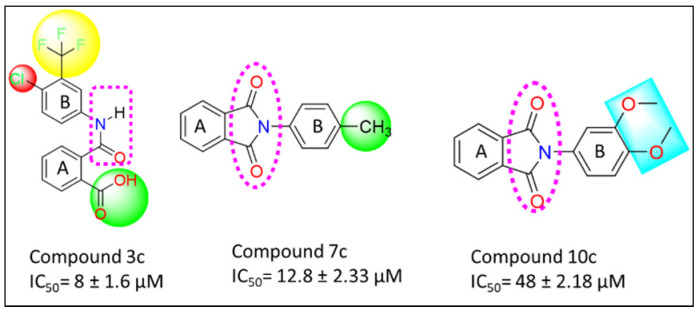
Structures and IC_50_ values of the synthesized compounds against hepatic cancer.

Compounds **3c**, **7c**, and **10** show the best response against the HEPG2 cancer cell line; their IC_50_ values are better than thalidomide (standard) at 8 ± 1.6, 12.8 ± 2.33 and 48.0 ± 2.18 µM, respectively (Figure 10). Compound **3c** has four halogen atoms, i.e., one chlorine and three fluorine substituents present at the para and meta positions of ring B. This makes compound **3c** more potent than the rest of the compounds. The carboxylic acid group present at the ortho position of the amide linkage at ring A also plays its role in anti-cancer activity. The NH- group of the amide linkage binds with the active site of cancerous cells to cause cell death. Here, three fluorine atoms play a vital role in compound **3c** because the chlorine and carboxylic acid groups are also present in compound **4c**. Compound **7c** also shows a better response than the others. It contains one methyl group at the para position of ring B; because the methyl group is electron-donating in its nature, it activates ring B, with the π-bonds of phenyl ring B being more available for binding. The five-membered ring present at the center of rings A and B has two carbonyl groups that also play a role in the inhibition or percent viability of the HEPG2 cancer cell line. Compound **10c** also shows a better response against hepatic cancer. Compound **10c** has two methoxy groups present at the meta and para position of ring B, which makes compound **10c** a potent inhibitor. Compounds **4c**, **8c**, and **12c** also show good responses against the HEPG2 cell line, as their %viabilities are 26.89 ± 1.05, 24.81 ± 4.6, and 26.89 ± 1.05 µM, respectively. Compounds **4c**, **8c**, and **12c** contain a chlorine atom and fluorine and antipyrine groups, respectively, attached to their ring B; these groups play a key role in the cell death of cancerous cells. Compounds **1c**, **2c**, **6c**, and **9c** show moderate responses against the HEPG2 cell line, as their %viabilities are 35.58 ± 3.65, 29.35 ± 0.72, 32.8 ± 4.4, and 36.3 ± 0.2 µM, respectively.

All of the reported results are the mean of three independent experiments, and those compounds showing > 50% inhibition were further analyzed for their IC50 values at different dilutions. The IC50 values were calculated by using nonlinear regression analysis and GraphPad Prism version 8. SEM = standard error mean.

## 4. Materials and Methods

All of the required chemicals, reagents, and solvents, including phthalic anhydride, amines, dichloromethane (DCM), dimethyl sulfoxide (DMSO), methanol, acetic acid, acetonitrile, acetone, distilled water, ethanol, n-hexane, and ethyl acetate were purchased from Sigma Aldrich (St. Louis, MI, USA), Daejung Korea (Busan, Republic of Korea), and Alfa Aesar (Havervill, MA, USA), and were used as received without further purification. The melting point of the synthesized compounds was checked by using the Stuart SMP20 apparatus. The FT-IR spectra of the synthesized compounds were recorded via the ATR method on a Vertex FTIR 70. Bruker Advance III HD 400 MHz and 100 MHz were used to record the ^1^H and ^13^C NMR spectra, respectively. CDCl_3_ and DMSO-d_6_ were used as solvents.

Synthetic Scheme

**Scheme 1:**
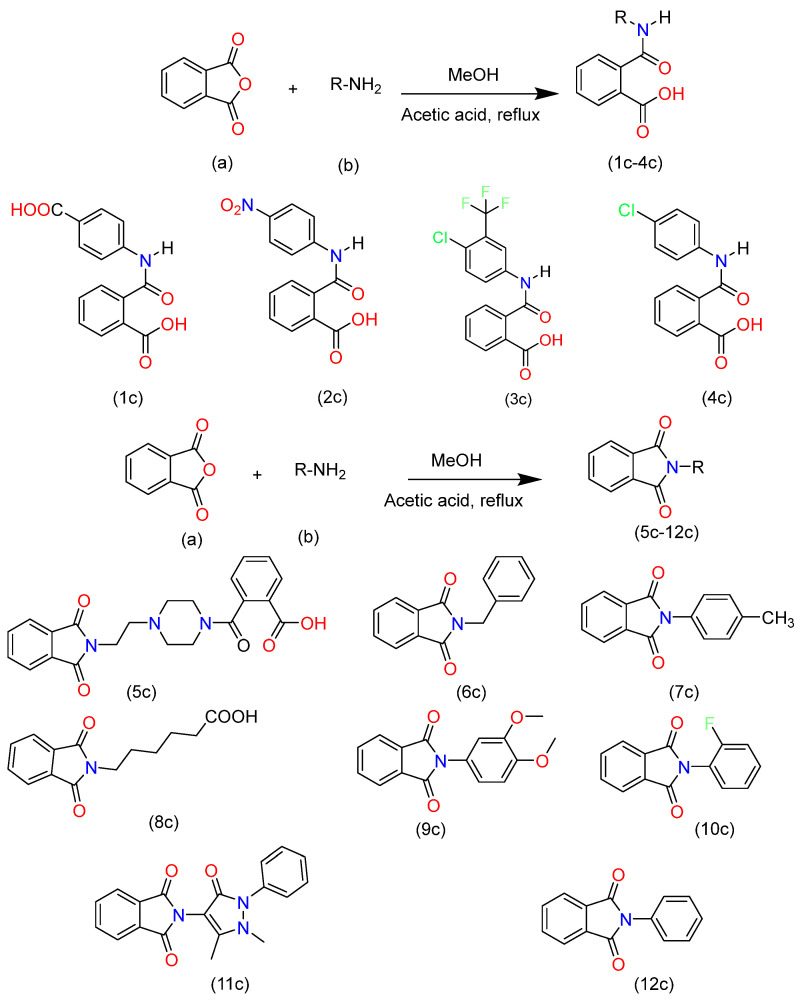
General synthetic route/scheme for phthalamic acid phthalimide derivatives.

Phthalic anhydride (a) was reacted with various substituted amines (b) in the presence of methanol and acetic acid; the reaction mixture was refluxed for 24 and 48 h, as illustrated in Scheme 1 respectively. After reaction completion, the reaction mixture was diluted with chilled water in order to obtain the precipitates (Scheme 1), followed by recrystallization, which resulted in the pure products: **1c**–**12c** [30].

Molecular Docking studies

MOE (molecular operating environment) [31] was used to investigate the binding interaction of the synthesized compounds with an active site of β-glucosidase. X-ray crystal structure of β-glucosidase (PDB IDs: 2XHY) was retrieved from the Protein Data Bank and was used as a starting structure for the docking and MD simulations [25]. Co-crystalized ligands and heteroatoms were removed, and docking was carried out using molecular operating environment—MOE 2016. The structures of the synthesized compounds were drawn by using Chem Office 3D (2015), and their energies were optimized using a Gaussian 09 employing B3LYP density functional theory method with a 6–311G basis set [32]. Initial optimization of the protein structure was carried out by using MOE software, followed by molecular docking studies. Eight of the lowest energy poses were generated for each compound, and the poses with the best binding energies were chosen for MD simulations. A Discovery visualizer (DSV) was used to determine the 2D and 3D models of the docked compounds.

### 4.1. Molecular Dynamic Simulations

Docked protein complexes and free protein were subjected to extensive MD simulation using periodic boundary conditions in explicit solvent systems. Initial protein structures were prepared using tleap, whereas the antechamber program was used to create the parameters for the compounds. All MD simulations were carried out using AMBER 18 with PMEMD.CUDA, employing General Amber Force Field (GAFF) and ff14SB force field for proteins and ligands. Each protein system was immersed in a TIP3P water box with at least 12 Å distances between the protein and the box edge in each direction. All hydrogen bonds were constrained by using the SHAKE algorithm and a simulation time step of 2 fs. Particle mesh Ewald (PME) was used to tackle long-range electrostatic interactions with an 8Å cut-off. The temperature and pressure of the systems were maintained using a Langevin thermostat and Berendsen barostat. The steepest descent and conjugate gradient approaches were used in series to gradually eliminate the restrictions before heating the system to stabilize it. Following minimization, each system was heated to 310 K for 500 ps by using the NVT ensemble. The system was subsequently relaxed by 500 ps in four phases in the NPT ensemble, with the restrictions gradually removed. After a series of equilibrations, the production runs for each system were carried out for 100 ns using the NPT ensemble. The trajectories obtained were analyzed in terms of RMSD, RMSF, MMPBSA analysis, and residue-wise energy contribution [33]. The anisotropic network model (ANMi) supported by the surface plot study (JMP 17) was used to identify the role of active loops in the functional dynamics of both the bound and free state of the target protein.

### 4.2. In-Vitro Studies

#### 4.2.1. Enzyme Inhibition Assay

The inhibition analysis of β-glucosidase was carried out, where *p*-nitrophenyl-β-D-glucopyranoside was used as a substrate, and the β-glucosidase from *E. coli* (purchased from Sigma Aldrich) was dissolved in buffer solution (pH 6.8). A total volume of 60 µL of sodium phosphate buffer, 10 µL of each test sample (dissolved in 1% DMSO), and 10 µL of enzyme (1.5 U/mL) were mixed together and incubated for 5 min at 37 °C, followed by the addition of 10 µL of *p*-nitrophenyl-β-D-glucopyranoside (dissolved in a buffer solution). Incubation of the mixture was carried out for 30 min, and 10 µL of 0.2 M sodium carbonate solution was subsequently added. DMSO (1%) was used as a negative control. The *p*-nitrophenol produced from *p*-nitrophenyl-β-D-glucopyranoside was measured at a λ_max_ of 405 nm in order to evaluate the activity of the test compounds against β-glucosidase [28]. The %inhibition of β-glucosidase was calculated by the following formula:

$$\%Inhibition=(100-\left[\left(\frac{sample}{control}\right)\right]\times 100$$

Graph Pad Prism v5.00 was used to find out the IC_50_ (concentration at which 50% of enzymatic activity was inhibited by the test sample) value from a second-order regression analysis.

#### 4.2.2. Cell Culture and Treatment

The human liver cells were cultivated in Dulbecco’s modified Eagle medium (DMEM), in which 100 U/mL penicillin, 10% fetal bovine, and 100 g/mL streptomycin were added and kept at 37 °C in a 50% carbon dioxide-induced humidified environment. The human liver cells prepared as per the above procedure were then treated with synthesized compounds diluted to a 0.05% DMSO concentration.

- **Determination of Cell Viability**

By using an MTT assay, the %viability of cancer cells [34] was evaluated. According to the standard procedure, the HepG2 cells were treated with various dosages of synthesized compounds for 48 h each. The cells were then incubated for 4 h at 37 °C with 10 μMTT reagent (5 mg/mL). Next, 150 μL of DMSO was added to dissolve the formazan crystals. The λ_max_ was recorded at 409 nm in a microplate reader (Thermos Scientific, Waltham, MA, USA).

## 5. Conclusions

NAFLD (non-alcoholic fatty liver disease) results in hyperglycemia, which eventually leads to liver cancer. Phthalimides and phthalamic acid derivatives are widely accepted as drug candidates against β-glucosidase and HepG2 cancer cells. Given the importance of this, the present study aimed to design new analogs of phthalimide and phthalamic acid to test their potential as inhibitors of β-glucosidase and the HepG2 cancer cell line by employing in silico and in vitro (β-glucosidase and HepG2 cancer cell line assays) approaches. The phthalimide and phthalamic acid analogs were synthesized in the presence of acetic acid and then characterized by using different spectroscopic techniques, like ^1^HNMR, ^13^CNMR, FT-IR, and Mass Spectrometry. In order to evaluate the potential of the synthesized compounds as leads against the target protein and HepG2 cancer cell lines, a combined enzyme inhibition assay, anti-hepatic cancer activity, and in silico studies were carried out using the standard protocols. By using molecular docking followed by MD simulations and free energy calculations, we were able to identify the binding mode of the compounds inside the active pocket, together with the role of individual residues, in facilitating the retention of the compounds in the binding pocket. It was observed that loops L1 and L2 form a lid over the active site, facilitating the closure of the binding pocket. Following this, enzyme inhibition assays were performed in order to check the inhibition potential of the selected compounds against β-glucosidase and the HepG2 cancer cell lines. All the compounds showed appreciable inhibition of β-glucosidase with an IC_50_ of 1.26 ± 0.23 µM, 1.26 ± 0.11, 2.17 ± 0.11 µM, and 3.00 ± 0.17 µM for **1c**, **5c**, **2c**, and **3c**, respectively. Compound **1c** had an IC_50_ value of 1.26 µM, which is better than the standard acarbose IC_50_ value of 2.15 µM against β-glucosidase. Compound **3c** was the most potent compound against the HepG2 cell lines, with an IC_50_ = 0.048 µM ± 0.67, when compared to standard thalidomide with an IC_50_ of 0.053 µM. Together, these findings pave the way toward the bioactivity-guided synthesis of a new class of phthalimide and phthalamic acid analogs that have the potential to be exploited for treating hepatic cancer and related diseases.

## Figures and Tables

**Table 1:** Docking scores and binding free energies computed from the MMPBSA study.

	∆G Kcal/mol	∆E_elec_	∆E_vdw_	∆E_GAS_ (elec + vdw)	PB-SOL (Polar + np)	∆G(Total) Kcal/mol
**1c**	−7.98	−6.27	−23.10	−29.37	11.14	−18.23
**2c**	−7.80	−7.04	−23.22	−30.26	12.70	−17.56
**3c**	−8.58	−7.21	−22.98	−30.19	10.12	−20.07
**5c**	−7.71	−6.52	−20.19	−26.71	11.37	−14.34

## Supplementary Materials

The following supporting information can be downloaded at https://www.mdpi.com/article/10.3390/molecules28186548/s1.

## Sample Availability

Samples of the compounds are available from the authors.
