# Supplementary material for: Bioactivity-Guided Synthesis: In Silico and In Vitro Studies of β-Glucosidase Inhibitors to Cope with Hepatic Cytotoxicity
Source: Molecules. 2023 Sep 9;28(18):6548. doi: 10.3390/molecules28186548 (PMC10538174; doi:10.3390/molecules28186548)
Supplement: Supplementary file 1 [file molecules-28-06548-s001.zip › molecules-2569233-supplementary.pdf]

Bioactivity-guided synthesis, in-silico and in-vitro studies of β-glucosidase inhibitors to cope with hepatic cytotoxicity

Aneela Khushal[a], Umar Farooq[a*], Sara Khan[a*], Azhar Rasul[b], Tanveer A. Wani[c], Seema Zargar[c], Sohail Anjum Shahzad[a], Syed Majid Bukhari[a], Nazeer Ahmad Khan[a]

[a]Department of Chemistry, COMSATS University Islamabad, Abbottabad Campus, KPK, Pakistan

[b]Department of Zoology, GC University Faisalabad, Pakistan

Table S1. physical data of synthesized compounds.

Sr. No.	Sample Code	Structure<br>Molecular formula<br>Molecular weight	M.P (°C)	TLC system & R_f	colour
**1.**	**Compound 1c**	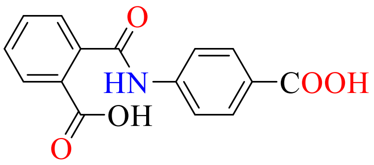	**290-292**	6:4 & 0.4	**White**
**2.**	**Compound 2c**	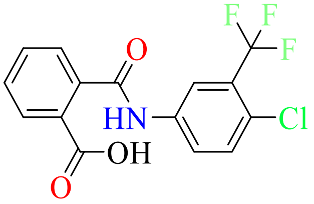<br>$C_{15}H_7ClF_3NO_2$ 325.67 g/mol	**200-203**	6:4 & 0.53	Off white
**3.**	**Compound 3c**	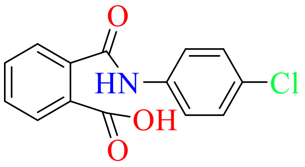<br>$C_{14}H_{10}ClNO_3$ 275.69 g/mol	**180-183**	6:4 & 0.4	White
**4.**	**Compound 4c**	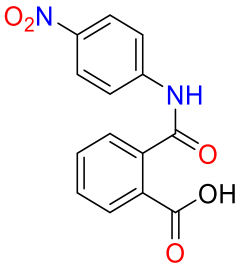<br>$C_{14}H_8N_2O_4$ 268.23 g/mol	265-268	8:2 & 0.35	Light green
**5.**	**Compound 5c**	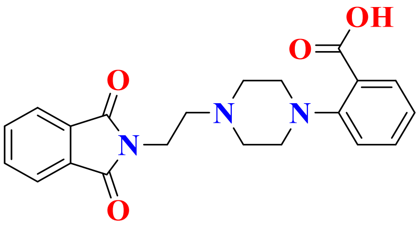<br>$C_{21}H_{21}N_3O_4$ 379.42 g/mol	235-245	7:3 & 0.55	White

**6.**	**Compound 6c**	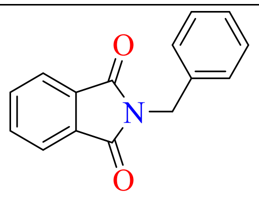 $C_{15}H_{11}NO_2$ 237.26 g/mol	135-138	9:1 & 0.7	White
**7.**	**Compound 7c**	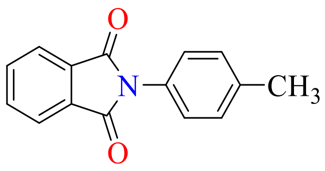 $C_{15}H_{11}NO_2$ 237.26 g/mol	200-202	7:3 & 0.53	White shiny crystal
**8.**	**Compound 8c**	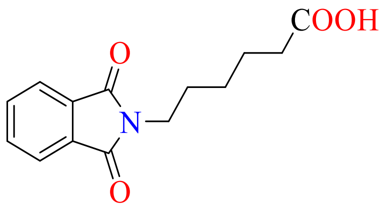 $C_{14}H_{15}NO_4$ 261.28 g/mol	155-157	8:2 & 0.4	Off white
9.	**Compound 9c/**	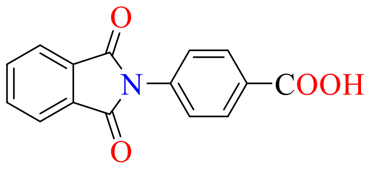			White
10.	**Compound 10c**	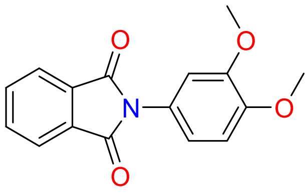 $C_{16}H_{13}NO_4$ 283.28 g/mol	285-295-	6.5:3.5 & 0.6	Light purple
11.	**Compound 11**	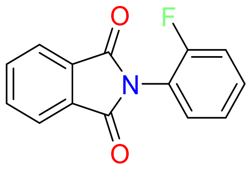 $C_{14}H_8FNO_2$ 241.22 g/mol	198-200	8:2 & 0.55	White shiny powdered

12.	**Compound 12c**	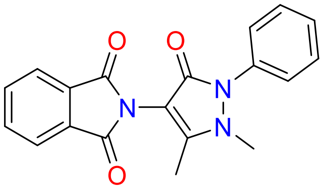 $C_{19}H_{15}N_3O_3$ 333.35 g/mol	290-292	9.5:0.5 & 0.45	light orange
13.	**Compound 13**	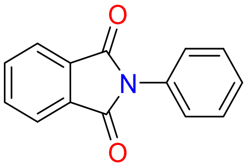 $C_{14}H_9NO_2$ 223.23 g/mol	278-280	3:7 & 0.49	White

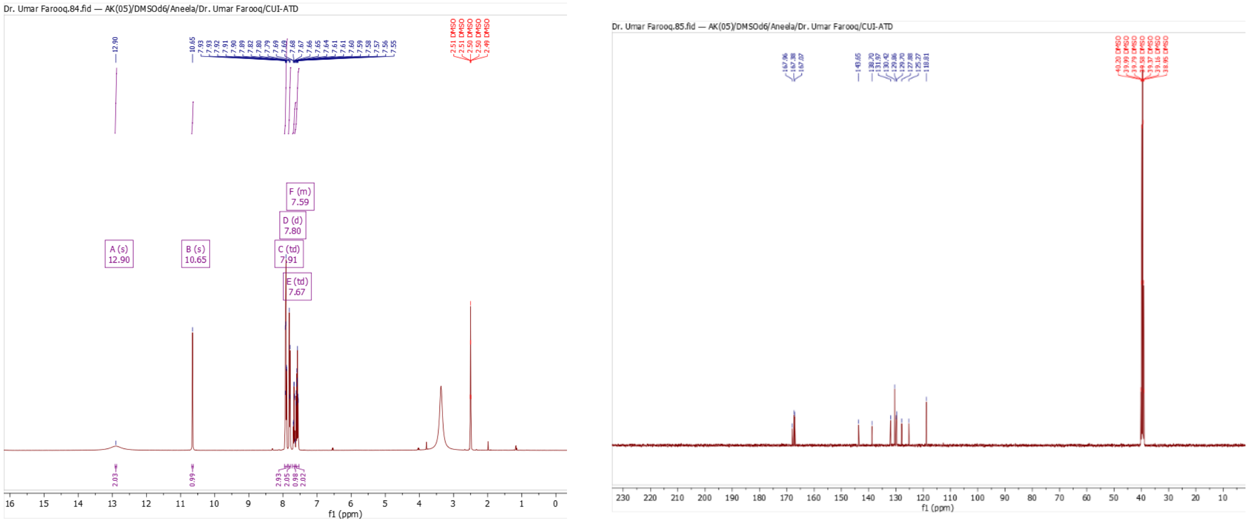

Supplementary Figure S1. ^{1}H and ^{13}C NMR spectra of compound 1C

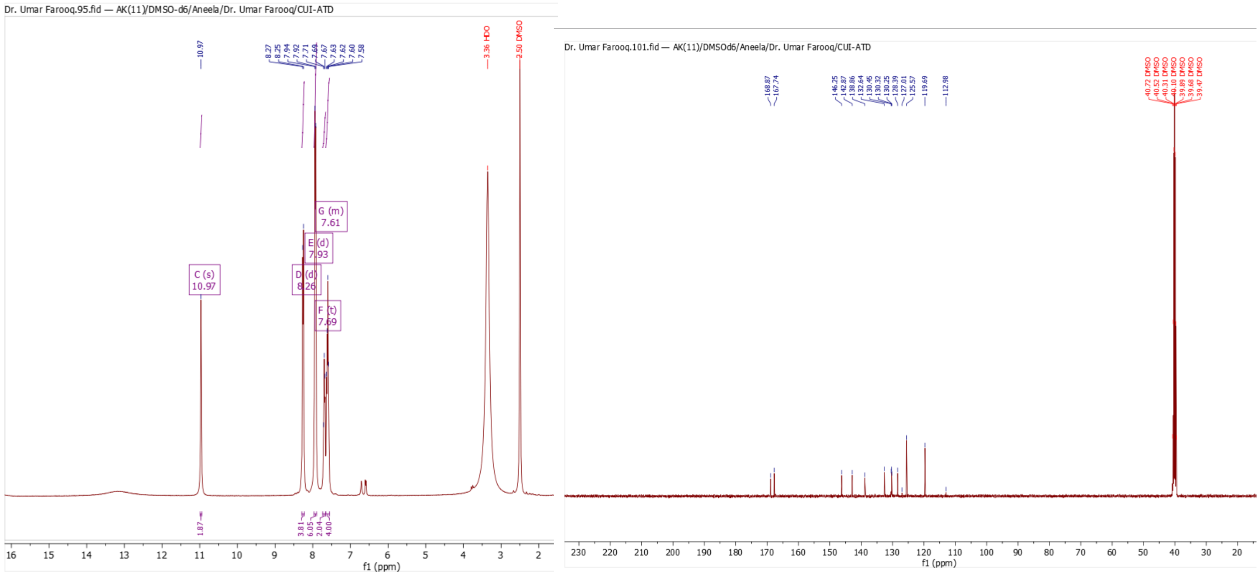

Supplementary Figure S2. ^{1}H and ^{13}C NMR spectra of compound 2C

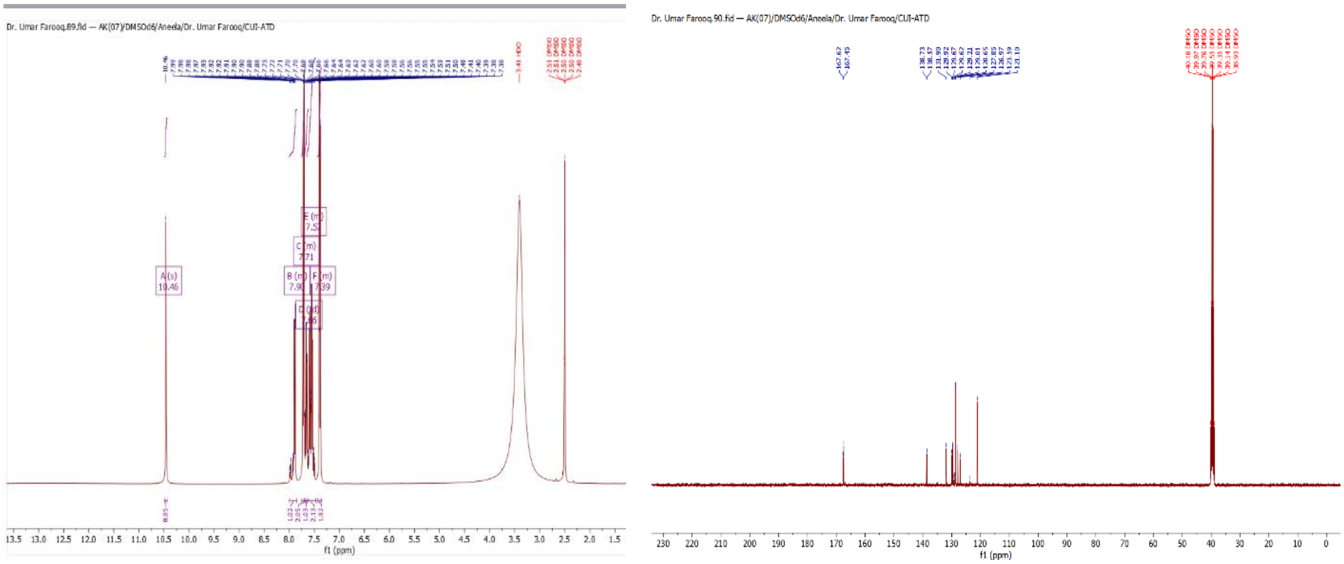

Supplementary Figure S3. ^{1}H and ^{13}C NMR spectra of compound 3c

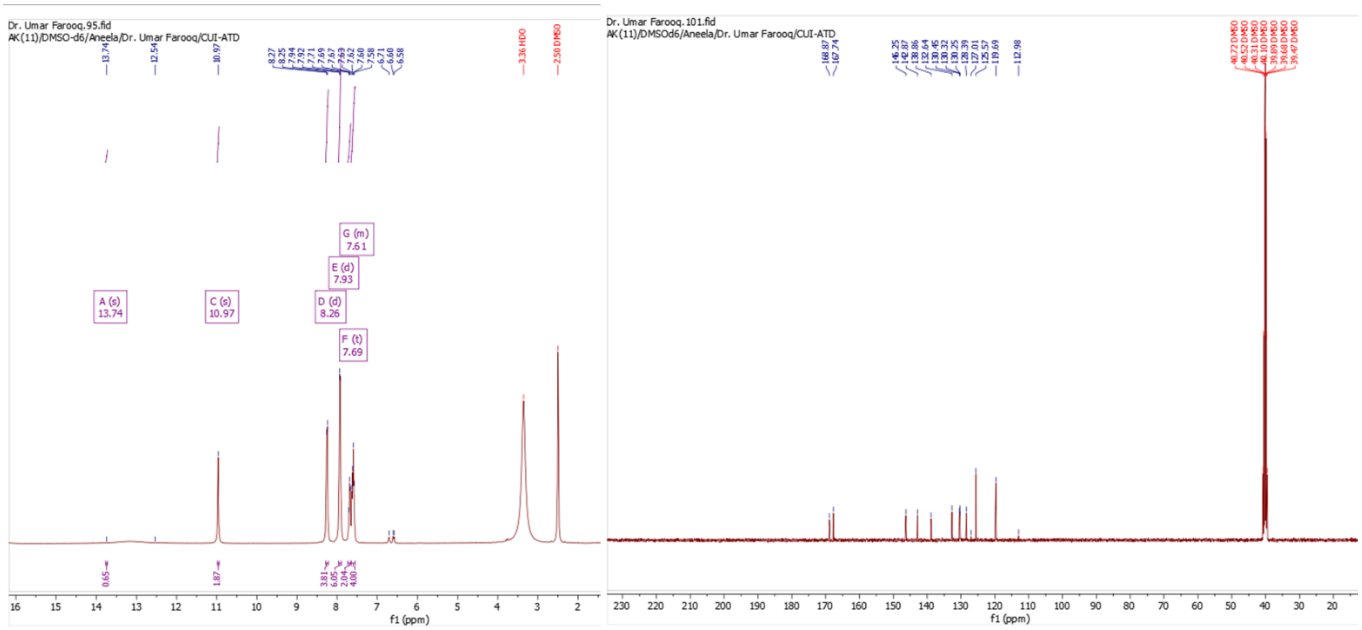

Supplementary Figure S4. ^{1}H and ^{13}C NMR spectra of compound 4c

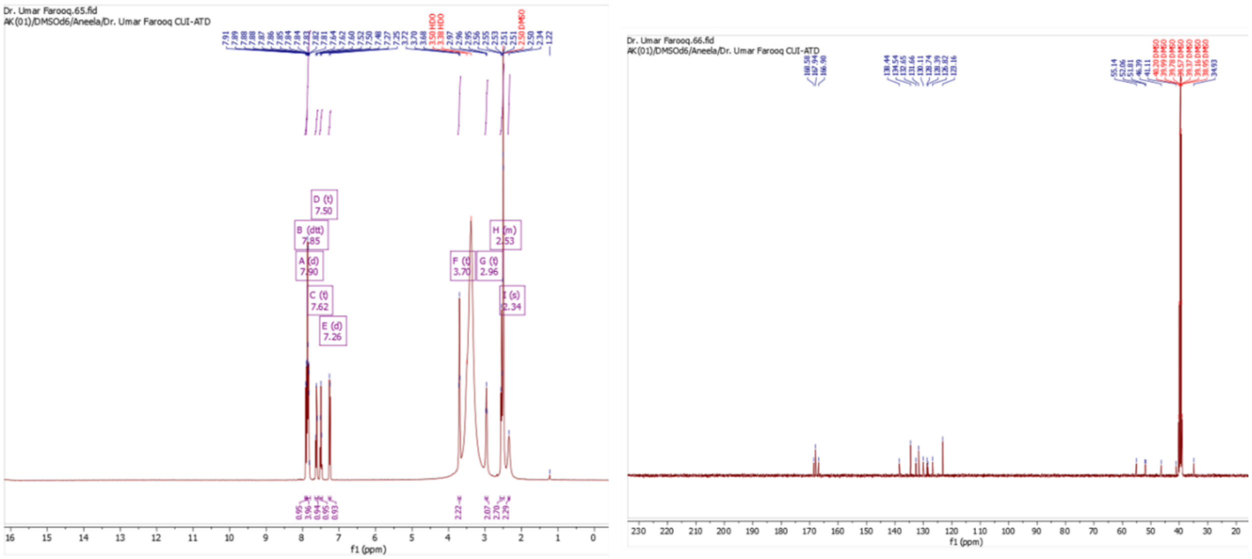

Supplementary Figure S5. 1H and 13C NMR spectra of compound 5c.

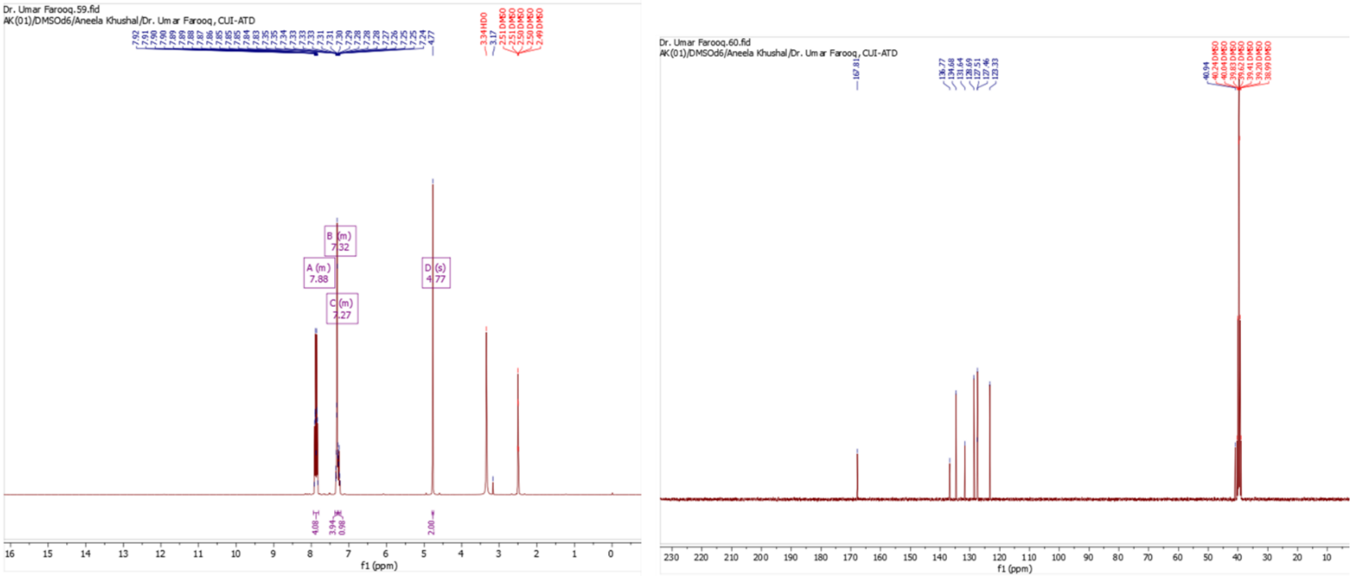

Supplementary Figure S6. ^{1}H and ^{13}C NMR spectra of compound 6c

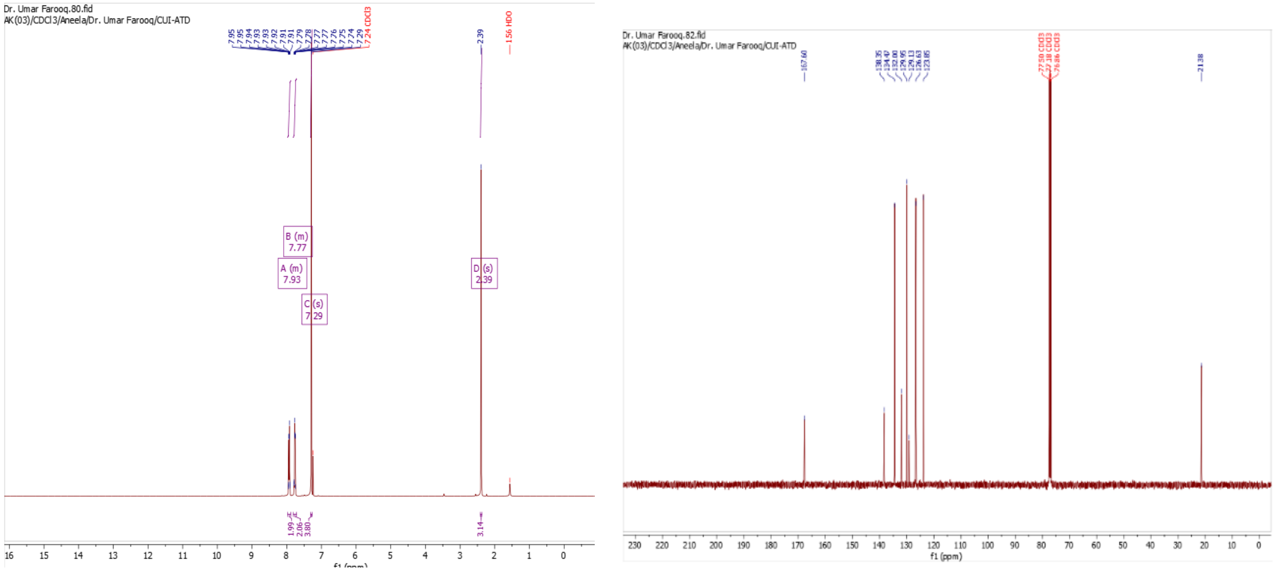

Supplementary Figure S7. ^{1}H and ^{13}C NMR spectra of compound 7c.

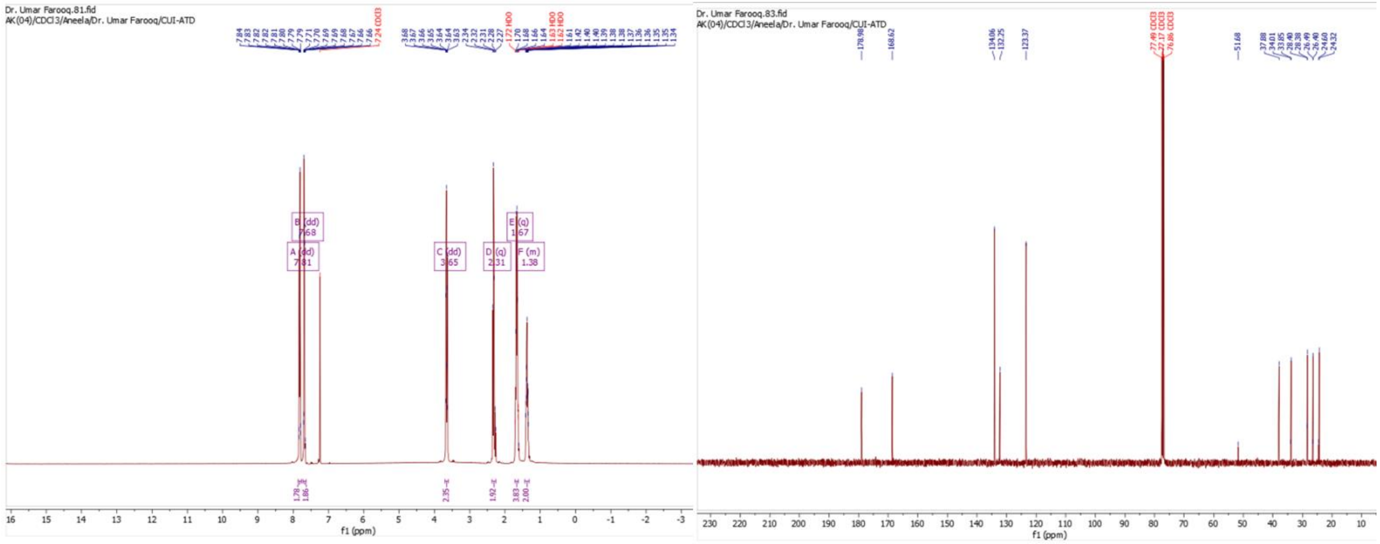

Supplementary Figure S8. ^{1}H and ^{13}C NMR spectra of compound 8c

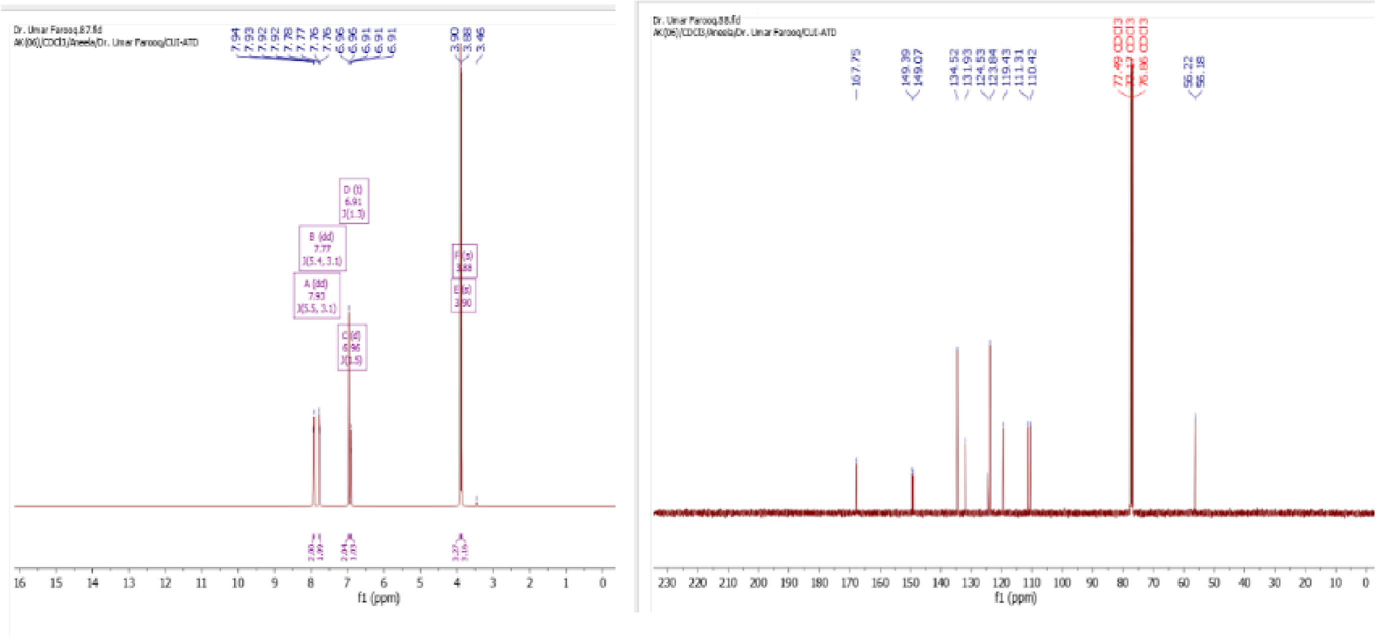

Supplementary Figure S9. ^{1}H and ^{13}C NMR spectra of compound 10c.

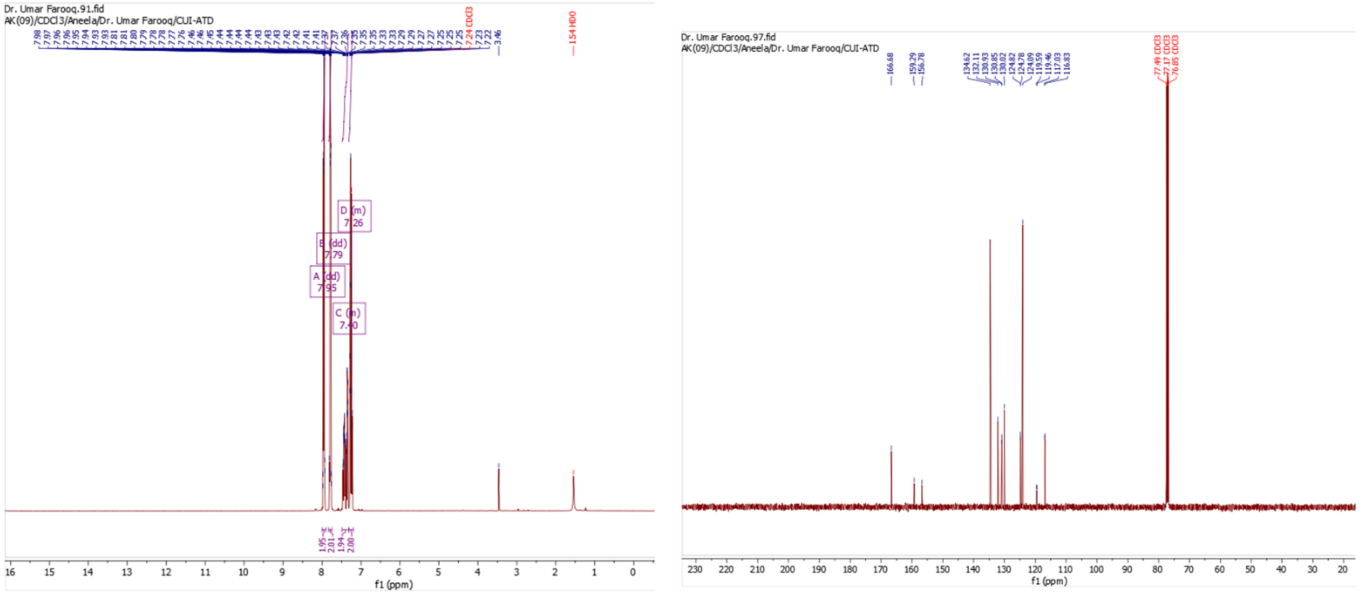

Supplementary Figure S10. ^{1}H and ^{13}C NMR spectra of compound 11c.

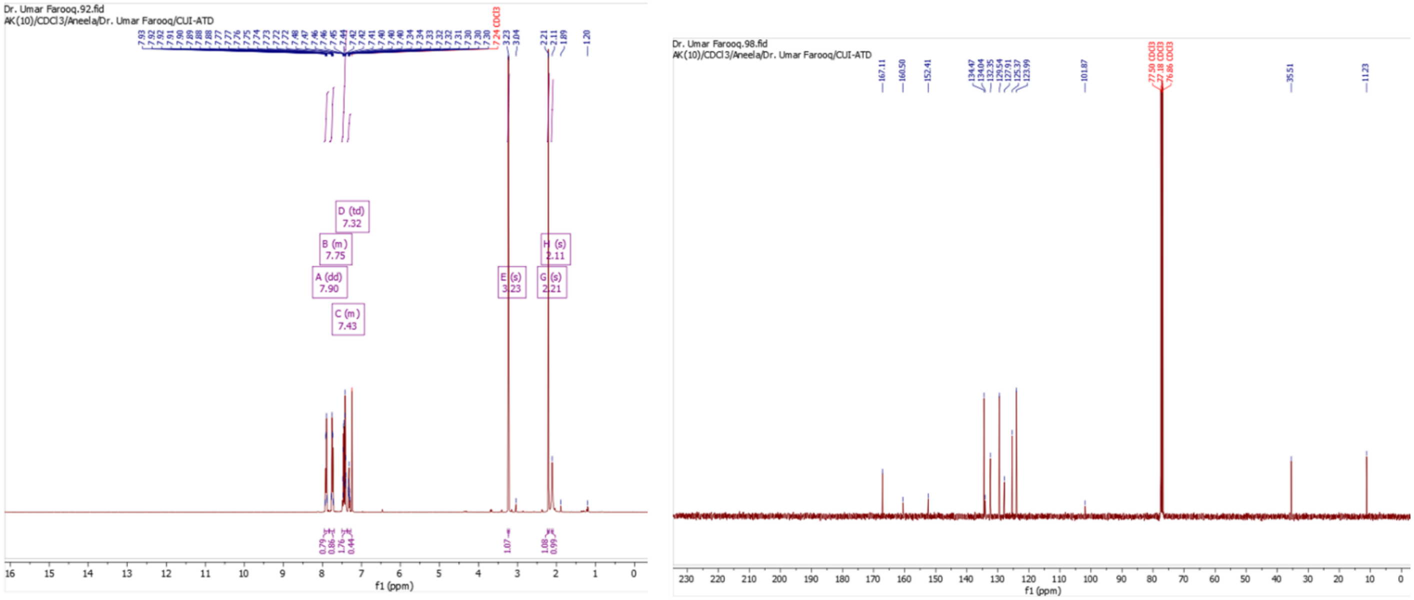

Supplementary Figure S11. ^{1}H and ^{13}C NMR spectra of compound 12c.

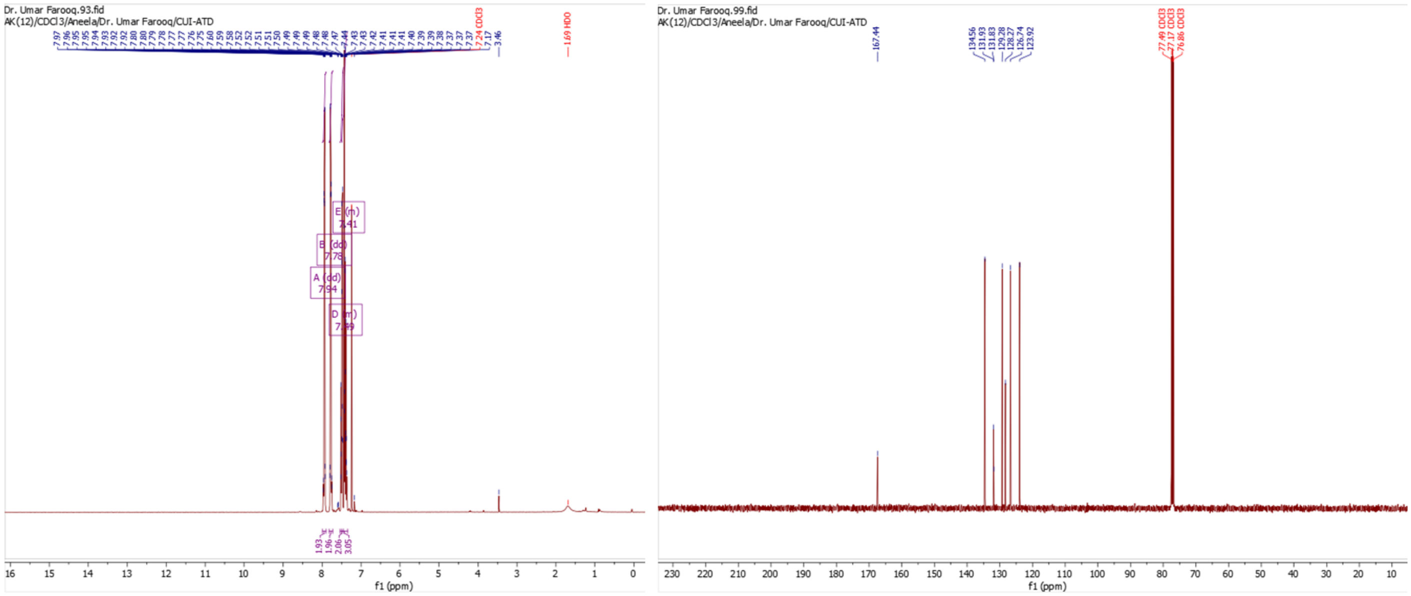

Supplementary Figure S12. ^{1}H and ^{13}C NMR spectra of compound 13c.

Supplementary Table S2. ADME score for compound1c-12c as computed from ProTox server

	Molecular weight	Number of rotatable bonds	Number of hydrogen bond donor	Number of hydrogen bonds acceptors	GI absorption	Lipinski rule violation	Bioavailability Score	PAINS alerts	Lead likeness violation
Compound 1c	285.25	5	5	3	High	0	0.56	0	0
Compound 2c	286.24	5	5	2	High	0	0.56	0	0
Compound 3c	343.69	5	6	2	High	0	0.85	0	1
Compound 4c	275.69	4	3	2	High	0	0.85	0	1
Compound 5c	379.41	5	5	1	High	0	0.55	0	1
Compound 6c	237.25	2	2	0	High	0	0.55	0	1
Compound 7c	237.25	1	2	0	High	0	0.55	0	1
Compound 8c	261.27	6	4	1	High	0	0.85	0	1
Compound 9c	267.24	2	4	1	High	0	0.55	0	0
Compound 10c	237.25	2	2	0	High	0	0.55	0	0
Compound 11c	283.28	3	4	0	High	0	0.55	0	1
Compound 12c	261.27	6	4	1	High	0	0.85	0	0

Supplementary Table S3. Residues facilitating the binding of compound 1c-3c within the active site of β-glucosidase.

**Sample**	ΔG kcal/mol
**1c**	-7.98
**2c**	-7.80
**3c**	-8.58
**4c**	-4.02
**5c**	-7.71
6c	-5.49
7c	-5.87
8c	-6.53
9c	-5.83
10c	-6.21
11c	-5.85
12c	-6.33
13c	-5.72

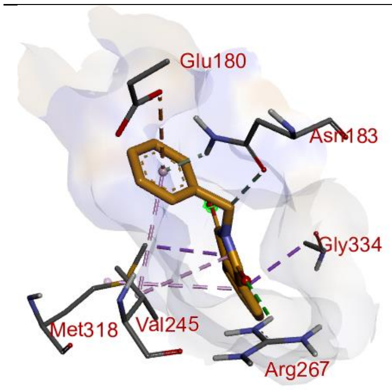
Glu180
Asn183
Gly334
Met318
Val245
Arg267

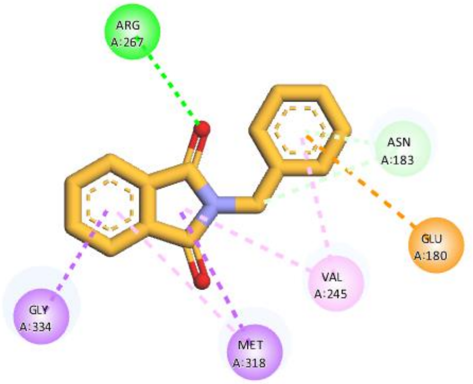
ARG A:267
ASN A:183
GLU A:180
VAL A:245
GLY A:334
MET A:318

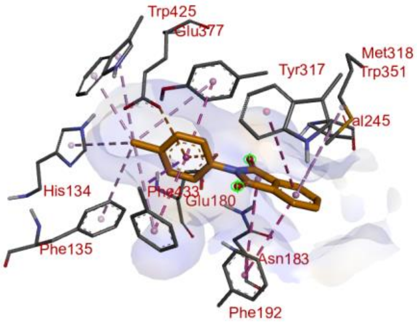
Trp425
Glu377
Met318
Trp351
Tyr317
Val245
His134
Phe433
Glu180
Phe135
Asn183
Phe192

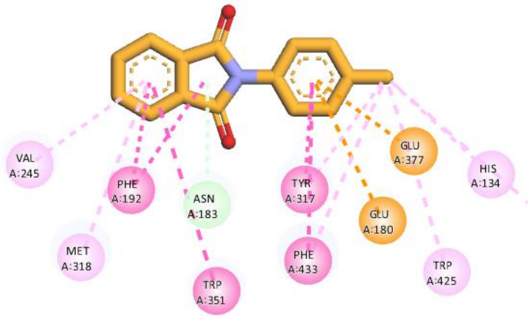
VAL A:245
PHE A:192
ASN A:183
TYR A:317
GLU A:377
HIS A:134
GLU A:180
MET A:318
TRP A:351
PHE A:433
TRP A:425

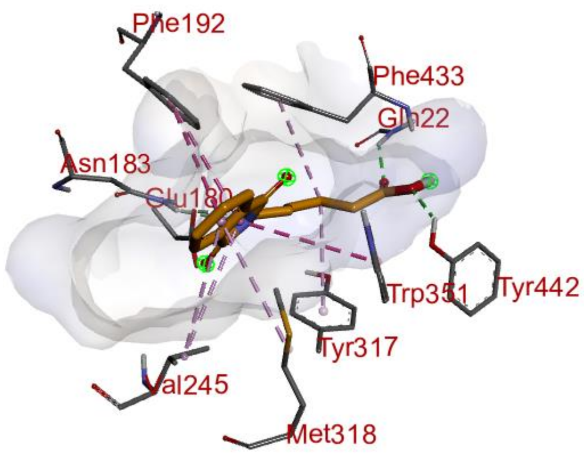
Phe192
Phe433
Gln22
Asn183
Glu180
Trp351
Tyr442
Tyr317
Val245
Met318

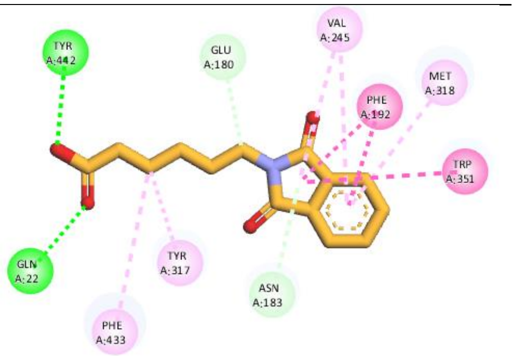
TYR A:442
GLU A:180
VAL A:245
MET A:318
PHE A:192
TRP A:351
GLN A:22
TYR A:317
ASN A:183
PHE A:433

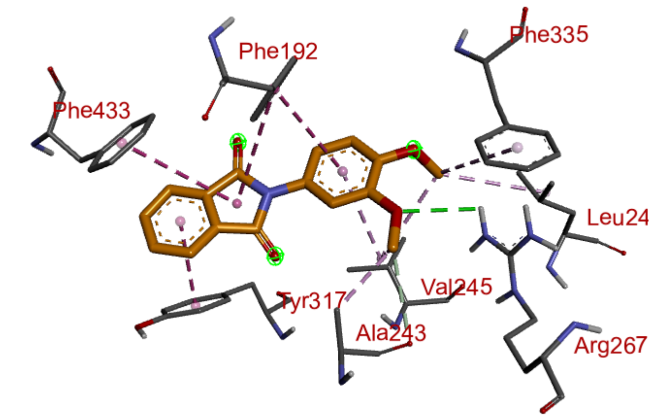
Phe192
Phe335
Phe433
Leu24
Tyr317
Val245
Ala243
Arg267

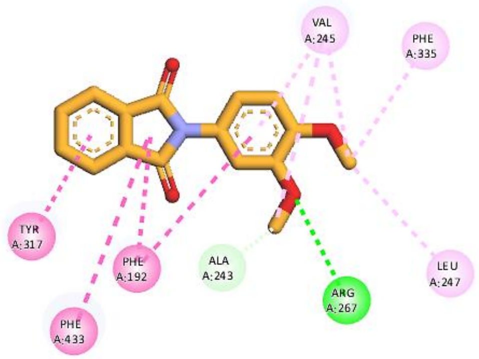
VAL A:245
PHE A:335
TYR A:317
PHE A:192
ALA A:243
LEU A:247
ARG A:267
PHE A:433

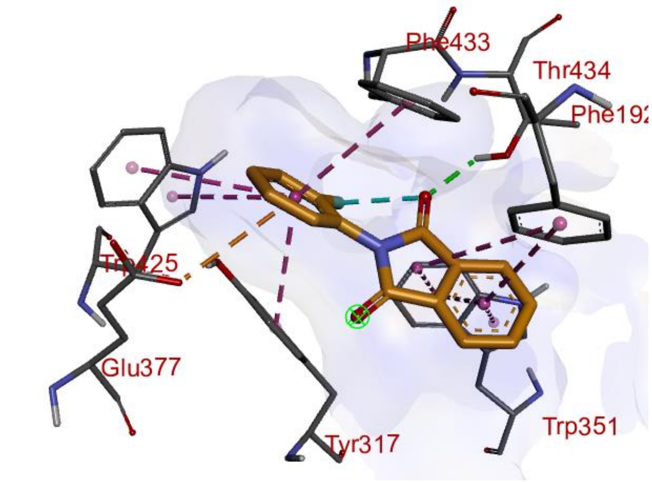
Phe433
Thr434
Glu377
Tyr317
Trp351

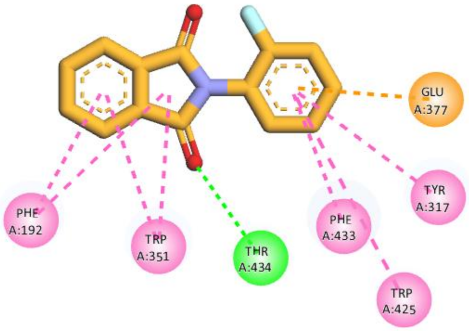
GLU A:377
PHE A:192
TRP A:351
THR A:434
PHE A:433
TYR A:317
TRP A:425

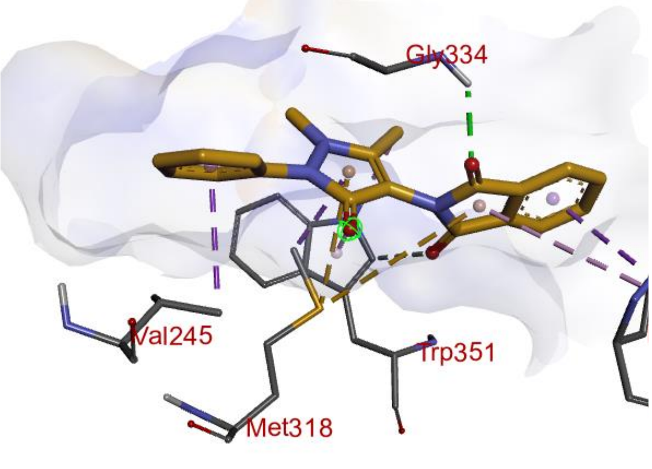
Gly334
Val245
Trp351
Met318

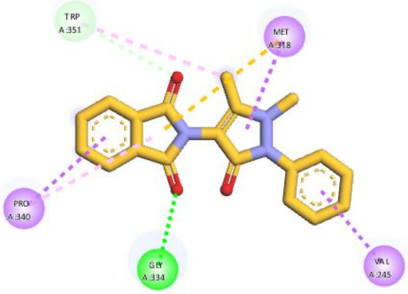

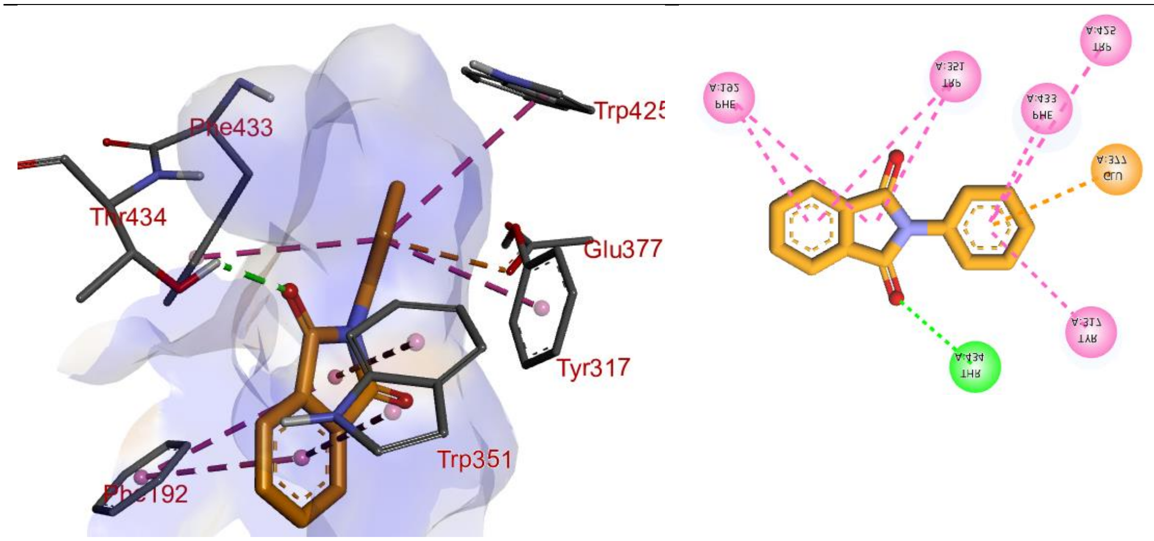

Supplementary Scheme S1. 2D and 3D binding interaction of compounds within the active pocket of β-glucosidase.

Supplementary Table S4. % inhibition and viability ± SD against β-glucosidase and HEPG2 cancer cell lines.

Sample	% inhibition ± SD against β glucosidase	IC_{50} ±SEM (μM)	% viability ± SD against HEPG2 cancer cell lines	IC_{50} ± SEM (μM)
1c	76 ± 1.41	1.26 ± 0.23	35.58 ±3.65	-
2c	68 ± 1.41	2.17±0.11	29.35 ±0.72	-
3c	70 ± 2.82	3.00± 0.17	12.77± 1.47	8± 1.6
4c	42 ± 1.42	-	26.89 ±1.05	-
5c	71 ± 1.2	1.26±0.11	42.22 ± 2.2	-
6c	45.0 ± 1.41	-	32.8±4.4	-
7c	63.5 ± 0.71	6.98 ± 0.31	21.41±3.4	12.8±2.33
8c	70.5 ± 2.12	4.78 ±0.27	24.81±4.6	-
9c	61.5 ±0.12	6.8 ±0.14	36.3±0.2	-
10c	50.5 ± 2.31	-	20.54±4.2	48 ±2.18
11c	56.5 ± 2.45	9.57 ±0.22	40.84±2.5	-
12c	45.2 ±0.4	-	26.89±1.05	-
Acarbose (standard)	74% ± 1.32	2.15± 0.16		

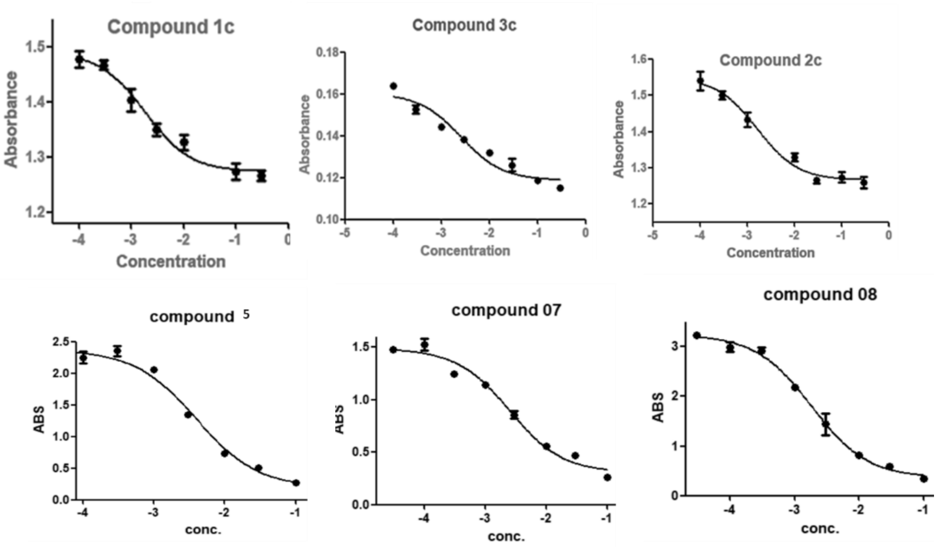

Supplementary Figure S13. IC_{50} graphs of compound 1c, 2c and 3c against *β*-glucosidase

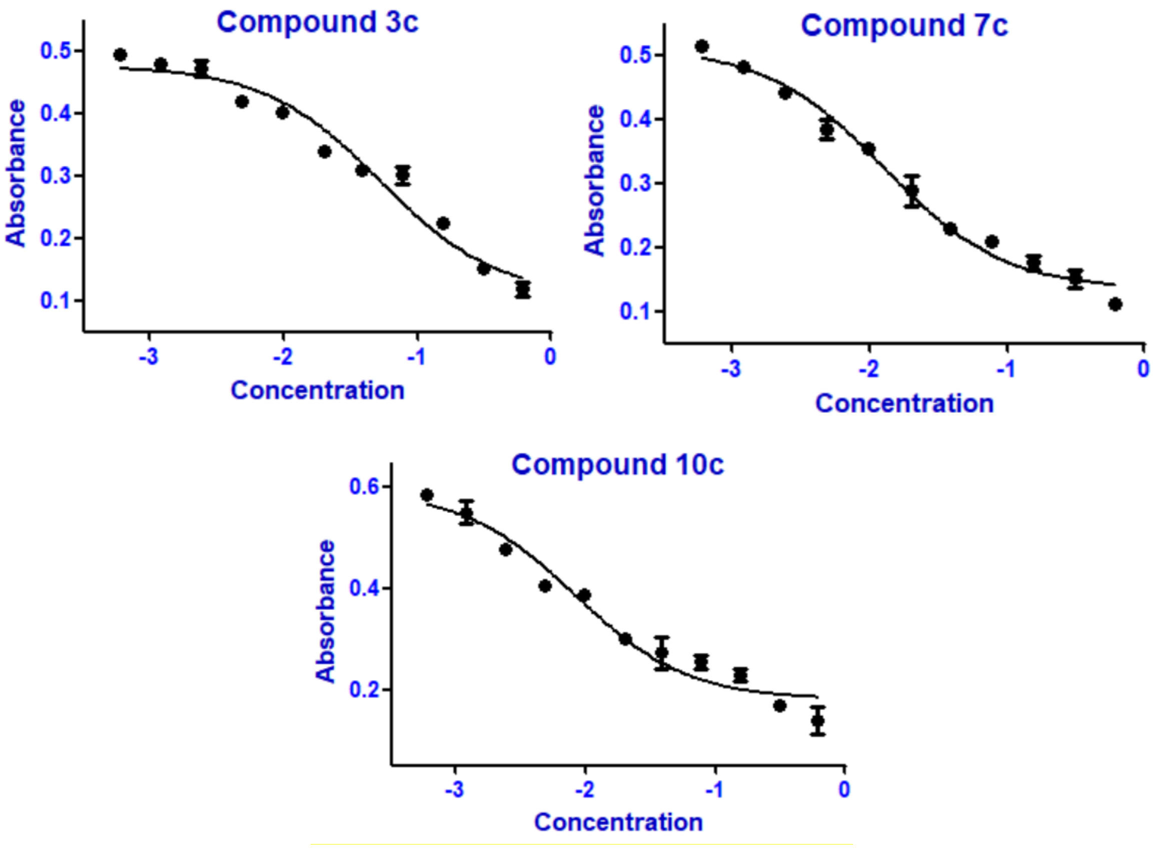

Supplementary Figure S14. IC_{50} graphs of compound 3c against HepG2 cancer cell.

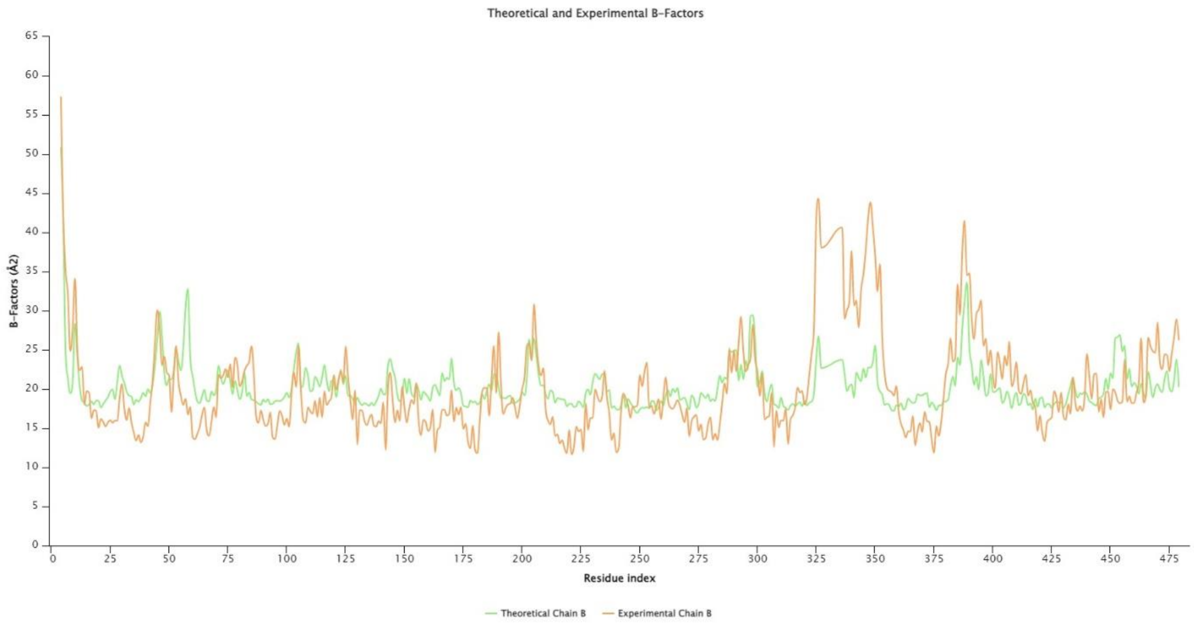

Supplementary Figure S15. theoretical and experimental B-factors of free protein.

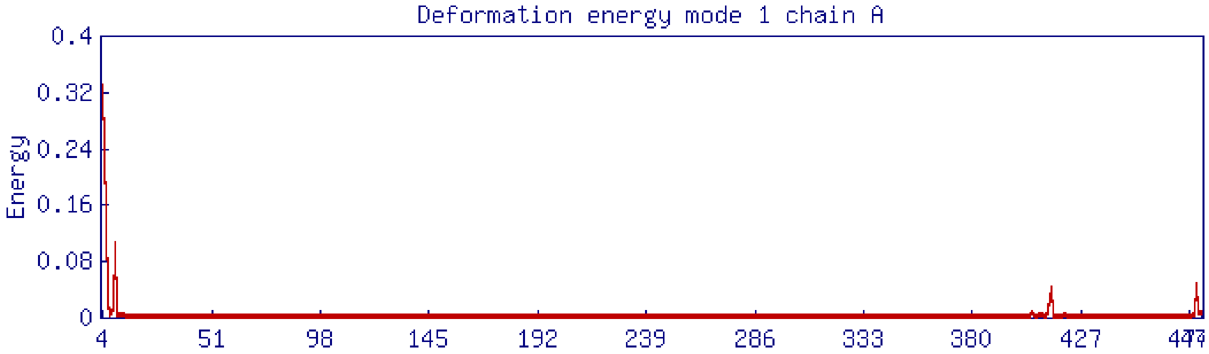

Supplementary Figure S16. Deformation energy of protein inhibitor complex.

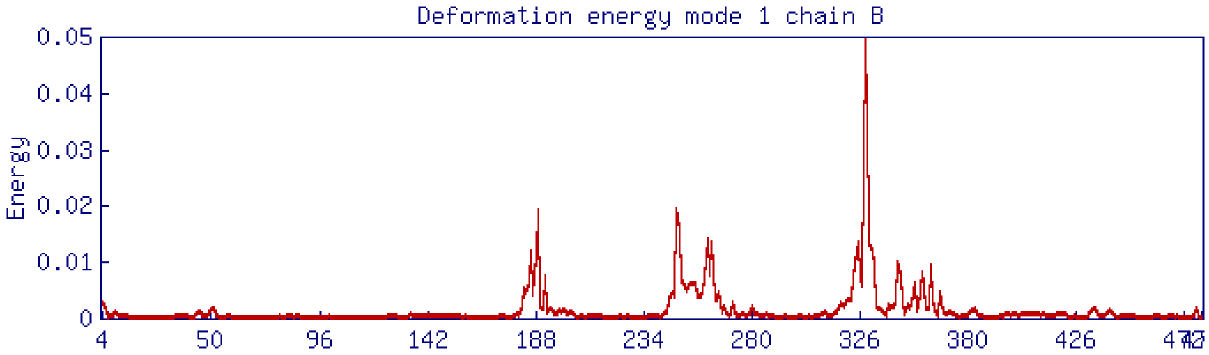

Supplementary Figure S17. Deformation energy of free protein.
